# Re-Establishment of the Genus *Ania* Lindl. (Orchidaceae)

**DOI:** 10.1371/journal.pone.0103129

**Published:** 2014-07-21

**Authors:** Lin Li, Hai-Fei Yan, Miao Niu, Tie-Yao Tu, Shi-Jin Li, Fu-Wu Xing

**Affiliations:** 1 South China Botanical Garden, Chinese Academy of Sciences, Guangzhou, China; 2 Graduate University of Chinese Academy of Sciences, Beijing, China; Field Museum of Natural History, United States of America

## Abstract

*Ania* Lindl. is a small genus of the tribe Collabieae subtribe Collabiinae (Orchidaceae). For the last 150 years, it has generally been treated as a synonym of *Tainia* Blume. In this study, we critically re-examined morphological characters that have been used to distinguish *Ania* from *Tainia,* and assessed the phylogeny of *Tainia* using morphological and palynological characters. Sequences of the nuclear ribosomal ITS, chloroplast *trnL* intron and combined DNA data sets were analysed to clarify the delimitation and the phylogeny of these groups. The morphological and palynological survey revealed a number of useful diagnostic characters which permit a clear definition of *Ania,* after the exclusion of a single taxonomically questionable species. Results confirmed that *Ania* is distinct from *Tainia*. Phylogenetic reconstructions based on molecular data provided the greatest resolution and produced a morphologically well differentiated clade of *Ania*. In addition to morphological and suggested palynological characters, the phylogenies were also supported by karyological evidence. Our results support the independent generic status of *Ania*. The genus name *Ania* is revived and re-established.

## Introduction

The acceptance of the genus *Ania* Lindl. (Orchidaceae) varied historically, with the majority of opinions tending to include it within *Tainia* Blume (see references [1]–[12]). The distinction between *Ania* and *Tainia* has historically not been clearly defined, and the delimitation of the genus *Tainia* sensu lato has been much debated. No comprehensive treatment is currently available for either genus. Given the present uncertainty about the relationships between these two groups, it seemed desirable to review the question in detail.

The genus *Tainia* was first described by Blume [13] and consists of about 28 species [14]–[15]. It occurs from India, China, and Southeast Asia to New Guinea, the Solomon Islands and Australia [11]–[12]. Blume [16] unnecessarily changed its generic name from *Tainia* to *Mitopetalum*. The genus *Ania* Lindl. was created by Lindley [17] based on two species collected in India. He did mention that the genus is characterized by a three-lobed, saccate or spurred lip. In the same work, however, Lindley included one species (*Ania latifolia*) without a spur on the lip [17]. Later, Blume [18] synonymised *Ania* under his genus *Mitopetalum*. Reichenbach *f.* [19] shared Blume’s opinion; he grouped Lindley’s *Ania* species under the former name *Taina*, which took precedence over *Mitopetalum*. Subsequent authors accepted the circumscription of *Tainia* as presented by Blume [13] and moved various *Ania* species to *Tainia* [1]–[4]. Then Ridley [20] came to a different conclusion. He separated some *Tainia* species in a new genus *Ascotainia* Ridl., which was characterized by having conical pseudobulbs, and free lateral sepals, not forming a mentum. This idea was rejected by Smith [5]–[6]. Schlechter [21]–[22] accepted both *Ascotainia* and *Tainia,* placing the two genera in two different subtribes; he considered that *Tainia belongs* in the Collabiinae and *Ascotainia* in the Phajinae. Tang & Wang regarded these two groups as independent genera. They made new combinations for certain species to resurrect the generic name *Ania*, but these new combinations have not been published until 1939 [23]. More recently, some authors have upheld the separation of *Tainia* and *Ania* [14], [24]–[26], while others have merged the two genera [7]–[12].

The generic delimitation of *Tainia* sensu lato is often inconsistent, which has caused confusion in taxonomic grouping. For example, many authors have stated that *Ania* differs generically from *Tainia* by having conical pseudobulbs and free lateral sepals [20]–[27]. Although most *Tainia* species have cylindrical pseudobulbs, some, such as *T. bicornis* Reichb. *f.*, have conical pseudobulbs. Furthermore, pseudobulbs become shrivelled and shrunk in a dried specimen; this make it difficult to differentiate between conical and cylindrical shapes.

In his monograph, Turner [14] followed those authors in separating these two genera, but he noted that he ‘failed to see in what way the lateral sepals of these two genera differ’. He utilized a combination of vegetative and reproductive characters, such as pseudobulb shape, leaf articulation, inflorescence position, and lip spur.

In an attempt to resolve the generic limits of *Ania* and the seemingly incongruent combinations of features shared with *Tainia* [15], we realized that these somewhat ambiguously circumscribed characters do not distinguish all species of *Ania* from *Tainia*. For example, the petiole is not articulated in *Ania viridifusca* (Hook.) T. Tang & F. T. Wang ex Summerh.; in *Tainia paucifolia* J. J. Smith the inflorescence is not terminal but lateral. It is undoubtedly clear that species in *Tainia* sensu lato include different elements.

In particular, an important problem in the delimitation of *Tainia* concerns the crucial differences between these two groups. As mentioned above, *Tainia* species possess a lip generally without spur. Most species previously referred to *Ania* by Turner [14] are characterized by a distinctly spurred lip, with the exception of *A. ponggolensis* A.L. Lamb ex H. Turner. Turner stated that this species is clearly different to the others. He provisionally placed it into *Ania* and pointed out its generic position is unclear. Apart from the major difference in lip, there are also several other differences. The question arose whether species with such significant differences could belong to the same genus. In addition, some floral characters, e.g., whether lateral sepals are free or not, have long been in dispute. A study that combining the morphological work with the analysis of molecular sequences is clearly needed, in order to identify which morphological characters correlate to the phylogenetic relationships.

In light of recent DNA phylogenetic analyses [15], [27], the monophyly of *Tainia* sensu lato began to be seriously challenged. However, the precise generic delimitation of these groups and their relationships remain largely unknown. In the present study, we present the molecular phylogeny using DNA sequence data based on both nuclear and plastid sequences, and reevaluate the morphological, cytological and palynological characters of *Tainia* sensu lato, in order to:

1. test the intrageneric classification of *Tainia* and construct its phylogeny based on both morphological and molecular data;
2. investigate whether *Ania* deserves recognition at generic rank;
3. gain a better understanding of the generic status, circumscription and taxonomic delimitation of *Ania* species.

This study is part of the ongoing research project of the revision of the species in the genus *Tainia*.

## Materials and Methods

### Ethics statement

The locations of the field studies are neither private lands nor protected areas. The species collected here are currently not included in the Chinese Red Data Book. The fieldwork was conducted under the valid permissions of the authority of each natural reserve, specifically Caiyanghe National Nature Reserve (Yunnan, China), Nanling National Nature Reserve (Guangdong, China) and Wuzhishan National Nature Reserve (Hainan, China). No specific permits are required for the present study.

### Taxon sampling and DNA extraction

Within *Tainia*, 17 samples representing 12 species in the genus were surveyed. The material for the phylogenetic analyses was carefully chosen so that the taxonomic sections of *Tainia* were well represented. As many taxonomically important species as possible were included in the analysis. In total, we assembled a dataset of 42 accessions (27 newly obtained and the remainder downloaded from GenBank) representing 34 species in 11 genera with an emphasis on sampling *Tainia* s.l. Representatives of *Eria* Lindl. and *Phreatia* Lindl. were selected as outgroups, guided by van den Berg et al. [28]. Based on evidence from recent molecular phylogeny [15], [27], we also included 19 representative species of the following genera within these major groups: *Acanthephippium* Blume ex Endl., *Ancistrochilus* Rolfe, *Calanthe* R.Br., *Cephalantheropsis* Guillaumin., *Collabium* Blume, *Nephelaphyllum* Blume, *Phaius* Lour. and *Spathoglottis* Blume. All taxa were collected in the field and grown in the greenhouse, resulting in silica-dried samples for all of them. Whenever it was possible, DNA was extracted from a minimum of two specimens with the typical characteristics of each taxon, to confirm its phylogenetic position. The material sampled for the molecular phylogeny associated with herbarium voucher specimens are given in Table S1 (see supplementary information).

Total genomic DNA was extracted from silica-gel dried leaf tissue following a modified 2×CTAB protocol [29]. The Polymerase chain reaction (PCR) amplifications were performed in PTC-200 thermocycler (Bio-Rad). We employed the nuclear marker of the internal transcribed spacer 1, the gene 5.8S, and internal transcribed spacer 2 (hereafter called ITS) and non-coding chloroplast region *trnL* intron. These two markers are phylogenetically informative at and above the species level for orchids [30]–[33].

For the nrITS region, we used the primers 17SE and 26SE of Sun et al. [34] and a PCR program consisting of an initial denaturation at 94°C for 3 min, followed by 35 cycles of denaturation at 94°C, annealing at 58°C for 1 min, and 2 min at 72°C extension, and a final extension for 5 min at 72°C. The reaction mix for ITS contained 0.3 µL (5 U/µL) AmpliTaq polymerase, ddH_2_O 16.7 µL, 10× buffer, (MgCl_2_) 2.5 µL (0.2 mmol/L) dNTP, 0.5 µL (10 µmol/L) of each primer, 2 µL DMSO (dimethyl sulfoxide) and 10 ng/L template DNA. For the chloroplast *trnL* intron, we used the primers *trn-c* and *trn-d*, based on Taberlet et al. [35]. The reaction mix for *trnL* intron contained 0.3 µL (5 U/µL) AmpliTaq polymerase, ddH_2_O 15.7 µL, 10× buffer, (MgCl_2_) 2.5 µL, 2 µL (2.5 mmol/L) dNTP, 0.5 µL (10 µmol/L) of each primer, 1 µL DMSO (dimethyl sulfoxide) and 10 ng/L template DNA. The reaction conditions were: a first period of denaturation at 94°C for 3 min, followed by 35 cycles at 94°C, annealing at 52°C for 30 s, and 1 min at 72°C extension, and a final extension at 72°C for 7 min. Amplified fragments were purified using a DNA gel cleaning Kit (TaKaRa), and bidirectional sequencing reactions were performed by Invitrogen Trading Shanghai Co., Ltd.

### Sequence alignment and phylogenetic analyses

Sequence fragments were assembled and edited using the Sequencher [36] software package. For each marker, DNA sequences were initially aligned using Clustal X v.1.83 [37] and adjusted manually using the software Se-Al v.2.0a11 [38]. When aligning non-coding DNA sequences, we followed the recommendation of Kelchner [39]. Regions in the alignment that appeared ambiguous for *trnL* intron were excluded from the analyses. These regions included poly-A and poly-T regions corresponding to positions 573–730 of the aligned sequences (158 positions).

The phylogenetic reconstruction of the sequences was performed by maximum parsimony (MP) in PAUP* verion 4.0b10 [40]. Each dataset was analyzed separately and then a simultaneous analysis was performed including both regions. Before combining the datasets, the incongruence length difference (ILD) test was conducted to assess data congruency using PAUP* and 10, 000 heuristic search replications including only parsimony-informative characters. The ILD test failed to identify significant conflict between the nrITS and the plastid datasets. We therefore subsequently analyzed these two datasets in combination.

For the MP analysis, heuristic searches were performed with 1000 random addition sequences replicates, followed by tree-bisection- reconnection (TBR) branch swapping. MulTrees and Collapse options selected, setting no upper limit for MaxTrees. All characters were treated as unordered and equally weighted, gaps were treated as missing data. A strict consensus tree [41]–[42] was generated from the most parsimonious trees (MPT). The Bootstrap percentages [43] for support of the resulting nodes calculated from 1000 replicates using a heuristic search, each with 10 random-addition replicates following by tree bisection-reconnection (TBR) swapping algorithm. Because too many trees were found for the *trnL* intron data, we kept no more than 1000 trees per replicate. The consistency (CI) and retention index (RI) were calculated to estimate homoplasy.

Phylogenetic analyses of the separate and combined data analyses were also conducted using Bayesian inference. We identified the best-fitting substitution model for each DNA region (ITS, *trnL* intron and combined dataset) through Modeltest v.3.7 [44], implementing the Akaike information criterion (AIC) to compare models. The general time-reversible nucleotide substitution model, with among-site rate variation modeled with a gamma distribution (GTR+G), was selected for the *trnL* intron marker and the combined data, GTR+G and a proportion of invariable sites (GTR+I+G) were selected for the ITS marker. For all three analyses, Bayesian inferences were conducted using MrBayes v.3.1.2 [45]. The Markov Chain Monte Carlo (MCMC) algorithm was run for 2,000,000 generations with four incrementally heated chains, starting from random trees and sampling one out of every 200 generations. The initial 25% of samples of each MCMC run were discarded as burn-in, after checking for stability on the log-likelihood curves, produced split frequencies of less than 0.01, showing convergence between the paired runs. The remaining trees were used to construct the Bayesian consensus tree.

Because parsimony and Bayesian inference may both be affected by long-branch attraction [46], the combined dataset was also analyzed using maximum likelihood (ML) in GARLI v.0.97 [47]. Models of evolution were specified, as described above for the BI analysis. Subsequently, 1000 non-parametric bootstraps were performed under the partition data mode.

### Morphological and palynological studies

Details of the flowers, particularly the pollinarium, were observed and photographed under a stereomicroscope (Zeiss DV4). Pollen morphology was analyzed using a Scanning-electron-microscope (SEM) type JSM-6360LV. The Morphological data used here have been gathered both by extensive observation and adaptation of descriptions from a number of taxonomic and review papers, as well as examination of herbarium specimens. These specimens were either from new field collections done for this study, or from various herbaria (AMES, BM, CAL, E, HITBC, IBK, IBSC, K, KUN, L, MO, NY, P, PE, SING, SZG, TAI, TAIF, US). Morphological data obtained from literature were confirmed with new observations as far as possible. Special care was taken to encompass the whole geographical range, and to evaluate the patterns of morphological variation within the species range, and their taxonomic utility. Observations of pollinaria were generally made from herbarium materials rehydrated in boiling water. In addition, pollinarium samples were also taken from the living plants cultivated in the greenhouse of South China Botanical Garden, CAS (SCBG). For SEM observations, pollinia were fixed in FAA (formalin–acetic acid–alcohol 10:5:50), dehydrated in an ethanol series, critical-point dried in liquid CO_2_ and sputter coated with gold for 5 min. To cover intraspecific variation, pollinia of 3–5 individuals per species were sampled.

In addition to traditional characters, we identified additional potentially useful characters that have previously been undescribed or overlooked, such as leaf vernation, width ratio of lateral sepals to petals, lateral sepal shape, column and column-foot length, column orientation, column wing, pollinium type and pollen surface (Table S2, characters 8, 26–27, 37–40, 44, 49–50). A total of 50 characters suitable for phylogenetic analysis were corded in the data matrix, consisting of 37 (10 vegetative and 27 reproductive) qualitative characters and 13 (4 vegetative and 9 reproductive) quantitative characters. The terminology mainly follows [7], [10], [48]. A descriptive list of morphological characters is presented in Table S2. Voucher specimens examined for detailed morphological analyses are listed in Table S3. Vouchers for representative species of palynological studies are listed in Table S4 (see Table S2–S4 in the Electronic Supplements).

The cladistic analysis was based on the morphological matrix (see Table S5) and performed using PAUP* version 4.0b10 [40] with a heuristic search strategy, TBR branch swapping, starting with 1000 random additions, all characters weighted equally, and the MULTREES option in effect. The analyses were repeated 1000 times with the random addition option. A strict consensus tree summarized sets of equally parsimonious trees. To assess confidence in clades, bootstrap (BS) tests were performed using 1000 bootstrap replicates with 10 random-addition replicates.

### Cytological study

Mitotic chromosomes were investigated using root tips taken from living plants cultivated in the greenhouse of SCBG (Table S4). Actively growing root tips were pretreated with 0.002 mol/L 8-hydroxyquinoline solution while shielded from light for about 1.5 h at 4°C, and then washed with distilled water, fixed in freshly prepared cold Carnoy’s Fluid (ethanol: acetic acid solution = 3:1) in a mixture of water and ice for 2–3 h at 4°C, washed with distilled water three times, and later hydrolyzed in 1 mol/L hydrochloric acid at 60°C for 7–8 min. After three more rinses in distilled water, root tips were stained in 1% aceto-orcein solution for more than 15 min, and then squashed on the slide for light microscopy. Chromosome counts were made using cells at metaphase division and observed under Zeiss Axioscope. Photographs were taken with a Zeiss Photomicroscope II. The chromosomes of at least ten metaphase cells were counted and the measurements of three cells were made.

## Results

### Molecular phylogeny

The aligned ITS dataset comprised a total of 853 bases, of which 310 were parsimony informative. The MP analyses produced 3 most parsimonious trees with the tree length of 1162, consistency index (CI) of 0.497 (excluding uninformative characters), and retention index (RI) of 0.738. The *trnL* intron dataset provided 1057 aligned bases (158 excluded), of which 93 were parsimony informative. Heuristic searches yielded 675 most parsimonious trees with the tree length of 309 steps, CI = 0.717 (excluding uninformative characters) and RI = 0.852. The main clades found in the parsimony analyses of the individual ITS and *trnL* intron datasets were generally congruent (see Fig. S1 and Fig. S2 in the Electronic Supplements). Compared with *trnL* intron sequence, the ITS region provided the higher number of parsimony informative characters, and the interspecific variation of the ITS sequence was higher than that of the *trnL* intron sequence. The combined ITS and *trnL* intron dataset consisted of 1910 aligned DNA bases (158 excluded), with a total of 403 parsimony informative characters. The MP analyses resulted in 3 most parsimonious trees of 1495 steps, with CI of 0.514 (excluding uninformative characters), and RI of 0.742.

**Figure 1:**
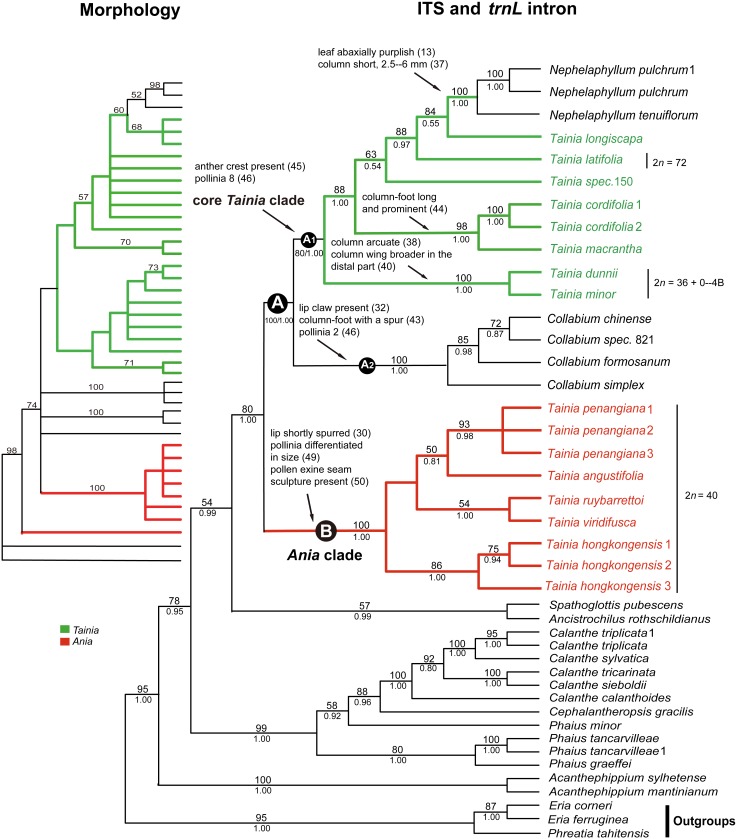
Phylogenetic relationships of *Tainia* s.l. among the main lineages. The cladogram on the left side represents a strict consensus tree based on morphological characters (see Fig. 4), the cladogram on the right side is the strict consensus tree of 3 most parsimonious trees resulting from the analyses of a combined dataset (ITS and *trnL intron*). Clades A and B indicate two main branches of *Tainia* s.l. A1, core *Tainia* clade. A2, *Collabium* clade. B, *Ania* clade. Numbers at the nodes indicate bootstrap values and posterior probabilities where support was ≥50 or BPP≥0.5. Numbers after a name differentiate multiple individuals sampled from one species. The delimitations for *Ania* and core *Tainia* are indicated by colored branches. Chromosome numbers are given for selected species.

In the analyses of the combined dataset, topologies generated by maximum likelihood (Fig. S3 in the Electronic Supplement) and Bayesian inference (not shown) are highly congruent with maximum parsimony (Fig. 1, right). Our molecular phylogeny indicated that all sampled members of *Tainia* in broad sense fall into two groups, clades A and B. Clade B comprises species previously referred to the genus *Ania*, forming a monophyletic clade with high bootstrap and Bayesian support (100% BS, 1.00 PP), which is strongly supported as sister (80% BS, 1.00 PP) to the clade A. Within the subclade A_1_, the core *Tainia* includes at least three major assemblages of species. The small clade (100% BS, 1.00 PP) that comprises sampled *Nephelaphyllum* is nested within *Tainia*, where it forms a well-supported sister group to the assemblages containing species *T. longiscapa.* The small clade (100% BS, 1.00 PP) that comprises a species previously assigned to the genus *Mischobulbum* [7]–[10], [12], [14]–[15], [21]–[22], [24]–[26] and its closely related species *T. macrantha* may also represent a distinct lineage of *Tainia* given further sampling. Within the subclade A_2_ (100% BS, 1.00 PP), *Collabium* is resolved as monophyletic with strong support.

### Morphology and palynology

**Figure 2:**
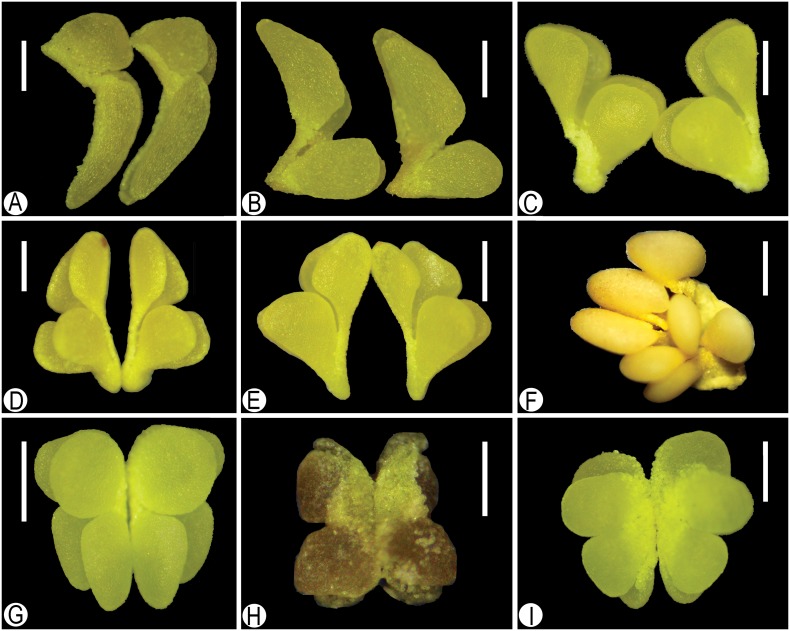
Representative LM photos of pollinarium types. **A–E,** pollinia differing in shapes or in sizes, and usually with two pairs of caudicles of unequal length; **F–I,** pollinia equal or subequal in shapes and sizes, with simillar caudicles: **A,** *Tainia angustifolia* (treated as *Ania angustifolia*); **B,** *T. hongkongensis* (treated as *A. hongkongensis*); **C,** *T. penangiana* (treated as *A. penangiana*); **D,** *T. ruybarrettoi* (treated as *A. ruybarrettoi*); **E,** *T. viridifusca* (treated as *A. viridifusca*); **F,** *T. macrantha*; **G,** *T. dunnii*; **H,** *T. minor* (pickled); **I,** *T. latifolia.* – Scale bars: 0.5 mm (A–E, G–I), 1 mm (F).

**Figure 3:**
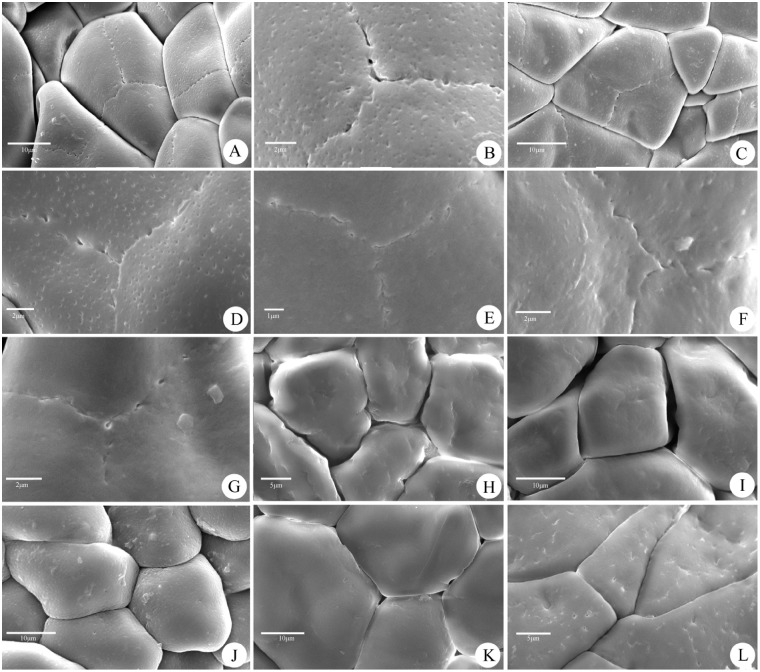
Representative SEM micrographs of pollen, showing variation in exine sculpture. **A–G,** laevigate or foveolate surface with seams; **H–K,** laevigate or foveolate surface without seams. **A–B,** *Tainia hongkongensis* (treated as *Ania hongkongensis*); **C–D,** *T. penangiana* (treated as *Ania penangiana*); **E,** *T. viridifusca* (treated as *Ania viridifusca*); **F,** *T. angustifolia* (treated as *Ania angustifolia*); **G,** *T. ruybarrettoi* (treated as *Ania ruybarrettoi*); **H,** *T. dunnii*; **I,** *T. latifolia*; **J,** *T. cordifolia*; **K,** *Nephelaphyllum pulchrum*; **L,** *Collabium chinense.* Arrows indicate seams. – Scale bars: 10 µm (A, C, I, J, K), 5 µm (H, L), 2 µm (B, D–G).

Previous studies dealing with the taxonomic position of *Tainia* did not consider the distinct discrepancy in their pollinaria. Morphological observations under the LM show significant variation in pollinaria of different species, with respect to the size, pollinia shape, and in the length of their caudicles (Fig. 2). In all samples examined under the SEM, pollen grains are irregular or polygonal in shape, 14.41–45.65 µm×12.16–23.58 µm in size, inaperturate, and occur in tightly packed tetrads. However, exine sculpture varies from laevigate, laevigate-porate, foveolate, regulate, to irregular perforate. The most eye-catching difference is the characteristic breaks or seams on the sporopollenin cap of outer tetrads of some species (Fig. 3A–G).

**Figure 4:**
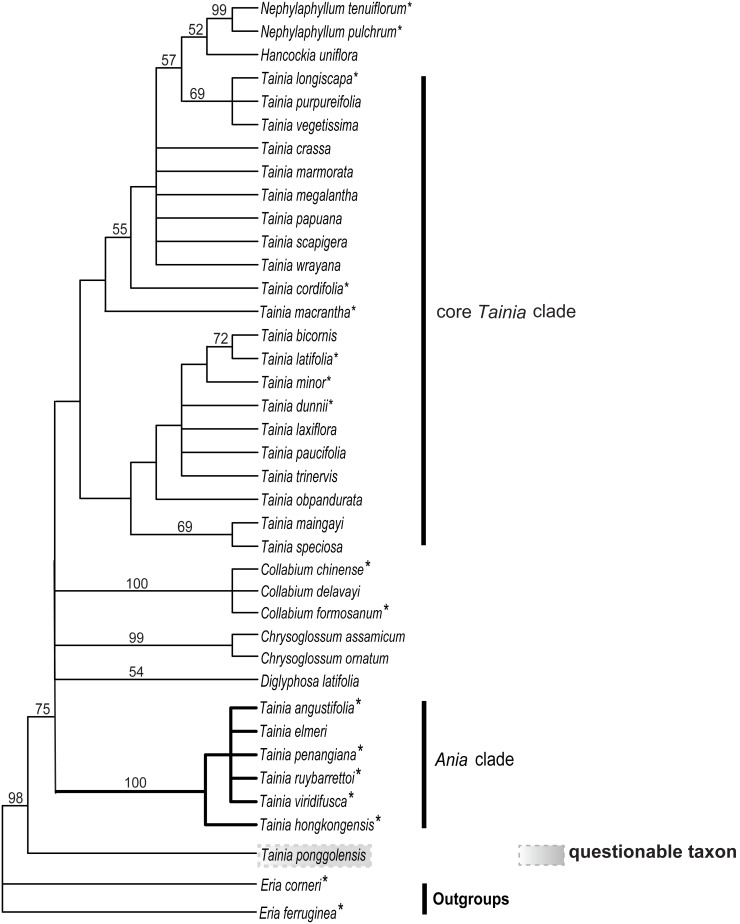
Strict consensus tree of 12 trees from parsimony analysis based on 50 morphological data (see Table S2) to show the placement of *Ania* and taxonomic sections of *Tainia*. Numbers above branches are bootstrap support (BS) values (>50%, 1000 replicates). A species previously classified under *Ania* is highlighted in gray as questionably placed. Highlighted branches are referred to in the text. Species included in the present molecular analyses are indicated with an asterisk.

Maximum parsimony (MP) analysis of the full matrix yielded 6 most parsimonious trees, with a length of 142 steps, CI = 0.497 (excluding uninformative characters); RI = 0.833. Of 50 total characters, 1 character is parsimony-uninformative, 49 characters are parsimony informative. The strict consensus of these trees is shown in Fig. 4.

Topology of the strict consensus tree based on morphological characters allowed us to identify several major clades within the ingroup. The cladograms indicate that *Tainia* s.l. is polyphyletic. Three *Tainia* species (*T. purpureifolia* Carr, *T. vegetissima* Ridl. and the related *T. longiscapa* (Seidenf. ex H. Turner) J.J. Wood & A.L. Lamb.) appear as sister to a clade composed by *Hancockia* Rolfe and representatives of *Nephelaphyllum* Blume with moderate support (60% BS). Morphologically, they share similar conduplicate, more or less fleshy, and abaxially purplish leaves. The clade consisting of species previously referred to *Ania* was supported as monophyletic with strong support (100% BS), after the exclusion of (*T. ponggolensis* (A.L. Lamb ex H. Turner) J.J. Wood & A.L. Lamb). Resolution was high and strong bootstrap support confirmed the monophyly of several traditionally recognized genera (e.g., *Collabium*, and *Chrysoglossum*.

### Cytology

**Figure 5:**
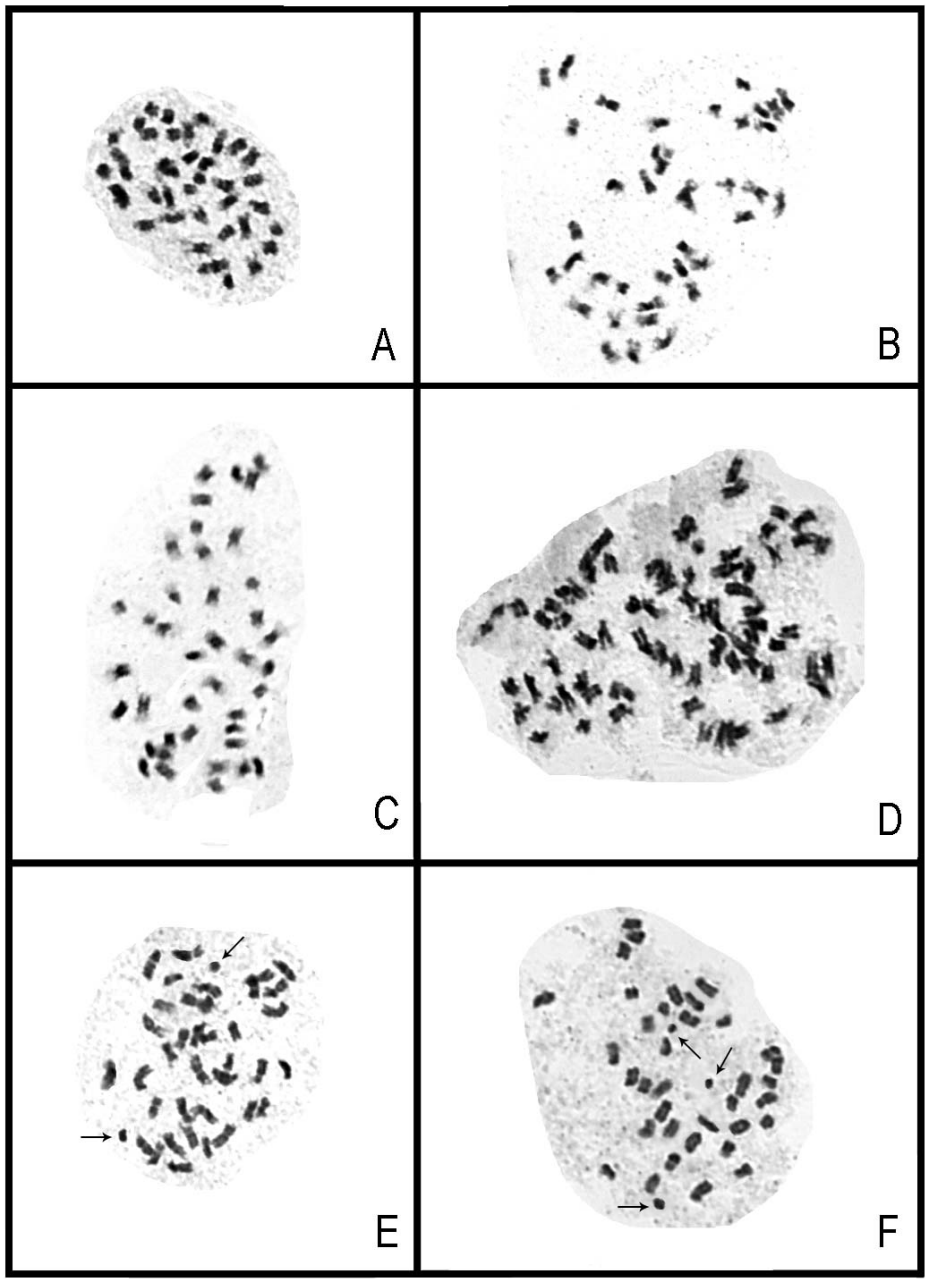
Micrograph of metaphase chromosomes in selected species of *Tainia* s.l. **A,** *T. penangiana* (treated as *Ania penangiana*); **B,** *T. hongkongensis* (treated as *Ania hongkongensi*); **C,** *T. ruybarrettoi* (treated as *Ania ruybarrettoi*); **D,** *T. latifolia*; **E,** *T. dunnii* (sampled from Guangdong); **F,** *T. dunnii* (sampled from Hainan). Arrows indicate B-chromosomes. – Scale bars = 10 µm.

The chromosome numbers in mitotic metaphase cells of the species investigated were counted to be 2*n* = 36+2B, 2*n* = 36+3B, 2*n* = 40, 2*n* = 72, respectively. In our cytogenetic analyses, B chromosomes were found in some species (Fig. 5E–F).

## Discussion

The overall topology generated from the molecular data is consistent with morphological data with respect to major groups, and in most cases strongly supported clades (Fig. 1). Morphological data helped to tentatively place several taxa for which DNA was not available. A summary of the morphological and cytological study is presented by mapping the most informative diagnostic characters at generic and species levels onto the phylogeny. These analyses indicate that all earlier delimitations of *Tainia* not only fail to meet the criterion of monophyly with respect to the analyzed morphological and sequence data, but demonstrate *Tainia* is clearly polyphyletic and requires taxonomic change at generic level.

The result of cladistic analysis based on morphological data confirms that the species of *Tainia* previously ascribed to *Ania* sensu Lindley [17] and Reichenbach [19] form a monophyletic group only if *T. ponggolensis* is excluded (Fig. 4). This delimitation of *Ania* has strong support in our molecular analyses and represents a morphologically clear-cut entity. The generic position of *T. ponggolensis* is doubtful. Morphologically this species possesses more than one leaf (liver-coloured when young), and has a pilose inflorescence of only two flowers with pilose sepals. It is the only species in *Ania* that has gourd-shaped pseudobulb, a distinctly long column-foot, and a lip without a spur. With the sole exception of this questionably-placed taxon, all other members of *Ania* share similar vegetative and floral features.

**Figure 6:**
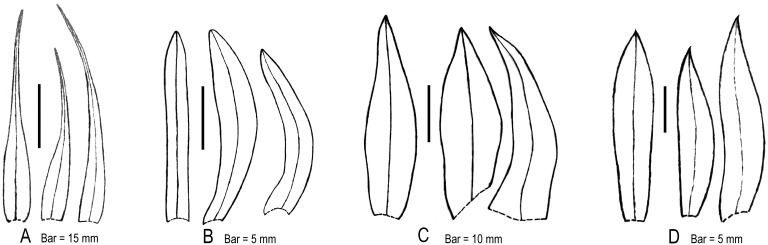
Tepal diversity of *Tainia* s.l. Left to right: dorsal sepal, petal, lateral sepal. **A–C,** Lateral sepals distinctly widened below the middle, widest at base, tapered to apex, more or less triangular or distinctly triangular, caudate-acuminate, petal broader than lateral sepal; **D,** Lateral sepal widest near middle, oblong-ovate, not or only slightly widened at base, not triangular, petal narrower than lateral sepal. **A,** *Tainia speciosa*; **B,** *T. latifolia*; **C,** *T. macrantha*; **D,** *T. ruybarrettoi* (treated as *Ania borneensis*).

**Figure 7:**
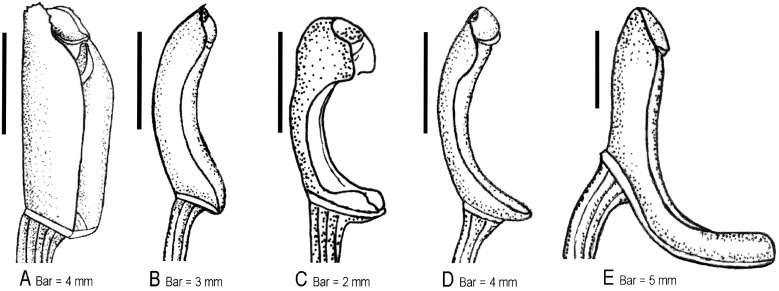
Column diversity of *Tainia* s.l. **A–B,** column porrect or slightly arcuate, stout (7–9 mm), with column wings present over total length, column-foot absent or indistinct, not more than 1.5 mm; **C,** column arcuate and stubby (3.5–6 mm), not more than 6 mm, distinctly arcuate, with column wings present only at the apex, column-foot around 1 mm; **D,** column distinctly arcuate, slender (7–9 mm), with column wings present only at the apex, column-foot 1.5–2 mm; **E,** column porrect and stout (10–11 mm), with column wings present over total length, column-foot long and prominent, *c*. 15 mm. **A,** *Tainia angustifolia* (treated as *Ania angustifolia*); **B,** *T. hongkongensis* (treated as *Ania hongkongensis*); **C,** *T. vegetissima*; **D,** *T. latifolia*; **E,** *T. macrantha.*

As mentioned above, traditional treatments used some vegetative characters such as pseudobulb shape to delimit the two genera, resulting in confused taxonomic grouping [20]–[23], [26]. Unlike a taxonomy based on vegetative characters, our morphological and palynological survey confirmed the diagnostic validity of some floral characters. For instance, flowers of all species formerly included in *Ania* (except for *Tainia ponggolensis*) are found with a spur at the lip base, whereas all other *Tainia* species do not develop a lip spur. Furthermore, our survey clearly revealed additional floral characters that are useful for genus and species delimitation. There is great diversity in the sepals and petals, which differ markedly in shape and size, and vary somewhat in the ratio of width of lateral sepals and petals (Fig. 6). Moreover, there are significant differences in the length of their column and column-foot (Fig. 7). These important floral differences are related to the phylogeny of the groups (Fig. 1), showing different diversification patterns within the clades.

Unfortunately, floral characters in this genus have been frequently confused. As stated above (see Introduction), whether or not the lateral sepals are free from the column-foot is controversial. In fact, as Turner [14] pointed out, the lateral sepals in both groups are slightly drawn out towards their base, and in both groups this outdrawn portion is curved downward and back around the column-foot, and thus in a sense the sepals are indeed adnate to column-foot. However, according to our observation, there are distinct differences in the length of the column-foot between the two groups (Fig. 7). Due to the long and remarkable column-foot, the lateral sepals in *Tania* appear to be adnate to, or broadly inserted on the column-foot, forming a distinct mentum. Our results indicate that the similarity between *Ania* and *Taina* is not as great as previously suggested. The short spur of *Ania* is only superficially similar to the mentum of *Tainia*, which is formed by the column-foot and the base of the decurrent lateral sepals. With respect to the comprehensive comparison, *Ania* are only linked with *Tainia* by homoplasies but not by true synapomorphies.

Palynological data obtained reinforce the distinction between *Ania* and *Tainia*. The observations under LM and SEM revealed that pollinarium type and pollen exine sculpture vary between different species, mostly in accordance with their sequence-based phylogeny, strongly suggesting a polyphyletic nature for the currently circumscribed *Tainia.* We investigated pollen morphology of five species of *Tainia* formerly included in *Ania* (5/7). They have pollinia distinctly different in size or shape, and usually have two pairs of caudicles of unequal length (Fig. 2). In addition, they develop a laevigate-foveolate surface with breaks or seams in the otherwise continuous sporopollenin cap of outer tetrads (Fig. 3). They are substantially distinctive in these characters with respect both to the species of *Tainia* and to the species included in the *Tainia*-*Nephelaphyllum* clade and *Collabium* clade. The current taxonomic circumscription lumps them all together.

The difference in the chromosome numbers between these two groups lends some support to the proposed systematic treatment of *Ania* as an independent genus. For all the samples formerly referred to *Ania*, the chromosome number in mitotic metaphase cells was counted to be 2*n* = 40. This result is in accordance with previous reports [49]–[52]. However, for the samples of *Tainia*, B chromosomes were found in *Tainia dunnii* Rolfe (Fig. 5, E–F) and different B chromosome numbers were found within each population of the same species, and counted as 2*n* = 36+2B; 2*n* = 36+3B, respectively. The existence of B chromosomes in *Tainia* has been reported previously. Tanaka & Matsuda [53] reported that some populations of *Tainia laxiflora* Makino have a high incidence (more than 95%) of individuals carrying B chromosomes in most of the clones. The chromosome number was 2*n* = 36+0–9B. Radhamoni et al. [54] found that *Tainia bicornis* (Lindl.) Reichb. *f.* possesses 2*n* = 30+3B chromosomes. Sharma & Chatterji [55] reported that the chromosome number of *Tainia minor* Hook. f. was 2*n* = 36 or 40; however, according to Radhamoni et al. [54], this was probably 2*n* = 36+0–4B. The patterns of different chromosome numbers of *Tainia* might have been obscured by the existence of B chromosomes. In addition, all the samples of *Tainia latifolia* showed polyploidy, with the chromosome number 2*n* = 72 (Fig. 5, D).

## Taxonomic Treatment

Based on the results of the molecular analyses and supported by distinctive morphological, palynological, and cytological characters presented in this paper, the re-establishment of the genus *Ania* is formally proposed here. *Ania* may be diagnosed by flowers with a spur at the base of the lip; lateral sepals broader than petals; column porrect or slightly arcuate; column-foot indistinct; and pollinia unequal in size or shape, with two pairs of caudicles of unequal length.

***Ania*** Lindey, *Gen*. Sp. Orch. 129. 1831; T. Tang & F.T. Wang ex Summerh., Bot. Mag. 161: t. 9553. 1939; T. Tang & F.T. Wang, Acta Phytotax. Sin. 1: 46, 88. 1951; A. Hawkes, Enc. Cult. Orch. 50. 1965; S. Y. Hu, Gen. Orch. Hong Kong 65. 1977; H. Turner in Orch. Monogr. 6: 49. 1992, p. p. maj., sp. *A. ponggolense* exc. – TYPE: *Ania angustifolia* Lindley (lectotype, designated by H. Turner in Orch Monogr. 6: 49. 1992).

 =  *Ascotainia* Ridley, Mater. Fl. Mal. Penin. 1: 115. 1907; J. J. Smith, Bull. Jard. Bot. Buit. II, 8: 6 (Sect. *Ascotainia*). – TYPE: *Ania penangiana* (Hook. f.) Summerh.

The following seven species are assigned to this genus.

***Ania angustifolia*** Lindley, Gen. Sp. Orch. 129. 1831 ≡ *Tainia angustifolia* (Lindl.) Benth. et Hook. f., *Gen*. Pl. 3: 515. 1883 ≡ *Ascotainia angustifolia* (Lindley) Schltr., Fedde Repert. Sp. Nov. Beih. 4: 246. 1919 (‘Ascotaenia’). – TYPE: Myanmar: Tenasserim: Tavoy, *Gomez s. n.* (holotype: K!; isotypes: BM!, E!).

 =  *Ascotainia sutepensis* Rolfe ex Downie, Kew Bull. 378. 1925 ≡ *Tainia sutepensis* (Downie) Seidenf. & Smitin., Orch. Thail. 2(1): 101. 1959. – TYPE: Thailand: Chiengmai: Doi Suthep, *A. F. G. Kerr 195* (holotype: K!).

 =  *Eulophia evrardii* Guillaumin, Bull. *Bot*. Fr. 77: 337. 1930 ≡ *Nephelaphyllum evrardii* (Guillaumin) T. Tang & F.T. Wang, Acta. Phytotax. Sin. 1: 77. 1951. – TYPE: Vietnam: Annam: west of Cana, *Evrárd 2387* (holotype: P!).

***Ania elmeri*** (Ames) A. D. Hawkes ex Senghas in Schltr., Die Orchideen 1 (ed. 3): 863. 1984 ≡ *Tainia elmeri* Ames, Elmer’s Leafl. Philip. Bot. 5: 1570. 1912 ≡ *Ascotainia elmeri* (Ames) Ames, Orchidaceae 5: 99. 1915. – TYPE: Philippine: Luzon: Mountain Province, *Elmer 8526* (not seen).

 =  *Tainia inamoena* Kränzlin, Fedde Repert. Sp. Nov. Beih. 17: 387. 1921. – TYPE: Philippine: Manila, *Loher 541* (holotype: CAL!).

***Ania hongkongensis*** (Rolfe) T. Tang & F.T. Wang, Acta Phytotax. Sin. 1: 46, 88. 1951 ≡ *Tainia hongkongensis* Rolfe, Kew Bull. 195. 1896 ≡ *Ascotainia hongkongensis* (Rolfe) Schltr., Die Orchideen 317. 1915, et Fedde *Repert*. Sp. Nov. Beih. 4: 246. 1919 (‘*Ascotaenia*’). – TYPE: China: Hong Kong, *C. Wright 522* (lectotype, designated by H. Turner in Orch. Monogr. 6: 54. 1992. K!; isolectotype: NY!, P!).

***Ania penangiana*** (Hook. f.) Summerh., Bot. Mag. 161: t. 9553. 1939 ≡ *Tainia penangiana* Hook. f., Fl. Br. Ind. 5: 820. 1890 ≡ *Ascotainia penangiana* (Hook. f.) Ridley, Mater. Fl. Mal. Penin. 1: 116. 1907; Schltr., Fedde Repert. Sp. Nov. Beih. 4: 246. 1919. (‘*Ascotaenia*’). – TYPE: Malaysia: Penang, on the top of the hill: *Maingay 1642* (holotype: K!; isotype: L!).

 =  ***Ania borneensis*** (Rolfe) Senghas, **syn. nov.** in Schltr., Die Orchideen 1 (ed. 3): 863. 1984 ≡ *Ascotainia borneensis* Rolfe in Gibbs, J. Linn. Soc. Bot. 42: 154. 1914. ≡ *Tainia rolfei* P. Hunt, Kew Bull. 26: 182. 1971. – TYPE: Malaysia: Borneo: Sabah, Mt. Kinabalu, *L. S. Gibbs 3958* (holotype: BM!; isotype: K!).

 =  *Ascotainia siamensis* Rolfe ex Downie, Kew Bull. 378. 1925 ≡ *Tainia siamensis* (Downie) Seidenf. & Smitin., Orch. *Thail*. 2 (1): 100. 1959. – TYPE: Thailand: Chiengmai: Doi Sutep, *A. F. G. Kerr 214* (holotype: K!).

 =  *Tainia hookeriana* King et Pantl., J. As. Soc. Beng. 64: 336. 1895 ≡ *Ascotainia hookeriana* (King et Pantl.) Ridley, Mater. Fl. *Mal*. Pen. 1: 116. 1907. – TYPE: Sikkim Himalaya, Teesta Valley, *R. Pantling 204* (holotype: BM!; isotypes: K!, P!).

 =  *Tainia malayana* J. J. Smith, Fedde Repert. Sp. Nov. Beih. 31: 76. 1932 ≡ *Ania malayana* (J. J. Smith) Senghas in Schltr., Die Orchideen 1 (3rd ed.): 863. 1984. – TYPE: Indonesia: Ambon: Goenoeng Toena, cult. in Hort. Bot. Bog., *J. J. Smith 109 m* (holotype: BO).

 =  *Tainia steenisii* J. J. Smith, Blumea 5: 306. 1943. – TYPE: Indonesia: Sumatra: Atjeh, Gajolanden, *Van Steenis 8960* (holotype: L! plant on right side).

***Ania ruybarrettoi*** S. Y. Hu & Barretto, Chung Chi J. 14: 25, t. 12. 1975 ≡ *Tainia ruybarrettoi* (S. Y. Hu & Barretto) Z. H. Tsi, Fl. Reip. Pop. Sin. 18: 243. 1999. – TYPE: China: Hong Kong, Mt. Tai Mo Shan, *S. Y. Hu 13098A* (holotype: K! isotype: PE!).

***Ania viridifusca*** (Hook.) T. Tang & F. T. Wang ex Summerh., Bot. Mag. 161: t. 9553. 1939 ≡ *Calanthe viridifusca* Hook., Bot. Mag. 78: t. 4669. 1852 ≡ *Tainia viridifusca* (Hook.) Benth. & Hook. f., Gen. Pl. 3: 515. 1883 ≡ *Ascotainia viridifusca* (Hook.) Schltr., Die Orchideen 317. 1915. – TYPE: India: Assam, introduced to Kew Garden, *Simon s. n.* (holotype: K!).

 =  *Calanthe eberhardtii* Gagnepain, Bull. Soc. Bot. Fr. 79: 162. 1932. – TYPE: Vietnam: Tonkin, Bac Kan, *Eberhardt 4673* (lectotype, designated by H. Turner in Orch. Monogr. 6: 60. 1992. P!).

## Supporting Information

Figure S1 **Strict consensus tree of 3 equally parsimonious trees inferred using nr DNA ITS dataset.** Bootstrap values are indicated above.
(PDF)

Figure S2 **Strict consensus tree of 675 equally parsimonious trees inferred using cp DNA** ***trnL*** **intron dataset.** Bootstrap values are indicated above(>50%).
(PDF)

Figure S3 **Maximum likelihood phylogeny of the combined datasets (ITS and** ***trnL*** **intron) with bootstrap values on the branches (>50%) to show the placement of** ***Ania*** **and the core** ***Tainia*** **.**
(PDF)

Table S1 **Voucher information and GenBank accession numbers of the sampled taxa in the molecular analysis.** Sequences generated in this study are marked with an asterisk (*).
(DOC)

Table S2 **Morphological characters included in the cladistic analysis (1–14, vegetative characters; 15–50, reproductive characters).**
(DOC)

Table S3 **Details of accessions included in the morphological study.**
(DOC)

Table S4 **Voucher information for representative species included in palynological and cytological study.** The number after the second hyphen in a voucher, e.g., “−1” indicates the individual in a sampled population.
(DOC)

Table S5 **Character matrix of taxa in cladistic analysis of** ***Tainia*** **and its related genera. ?  =  missing.**
(DOC)
