# Supplementary material for: Re-Establishment of the Genus Ania Lindl. (Orchidaceae)
Source: PLoS One. 2014 Jul 21;9(7):e103129. doi: 10.1371/journal.pone.0103129 (PMC4105443; doi:10.1371/journal.pone.0103129)
Supplement: Figure S1 — Strict consensus tree of 3 equally parsimonious trees inferred using nr DNA ITS dataset. Bootstrap values are indicated above. (PDF) [file pone.0103129.s001.pdf]

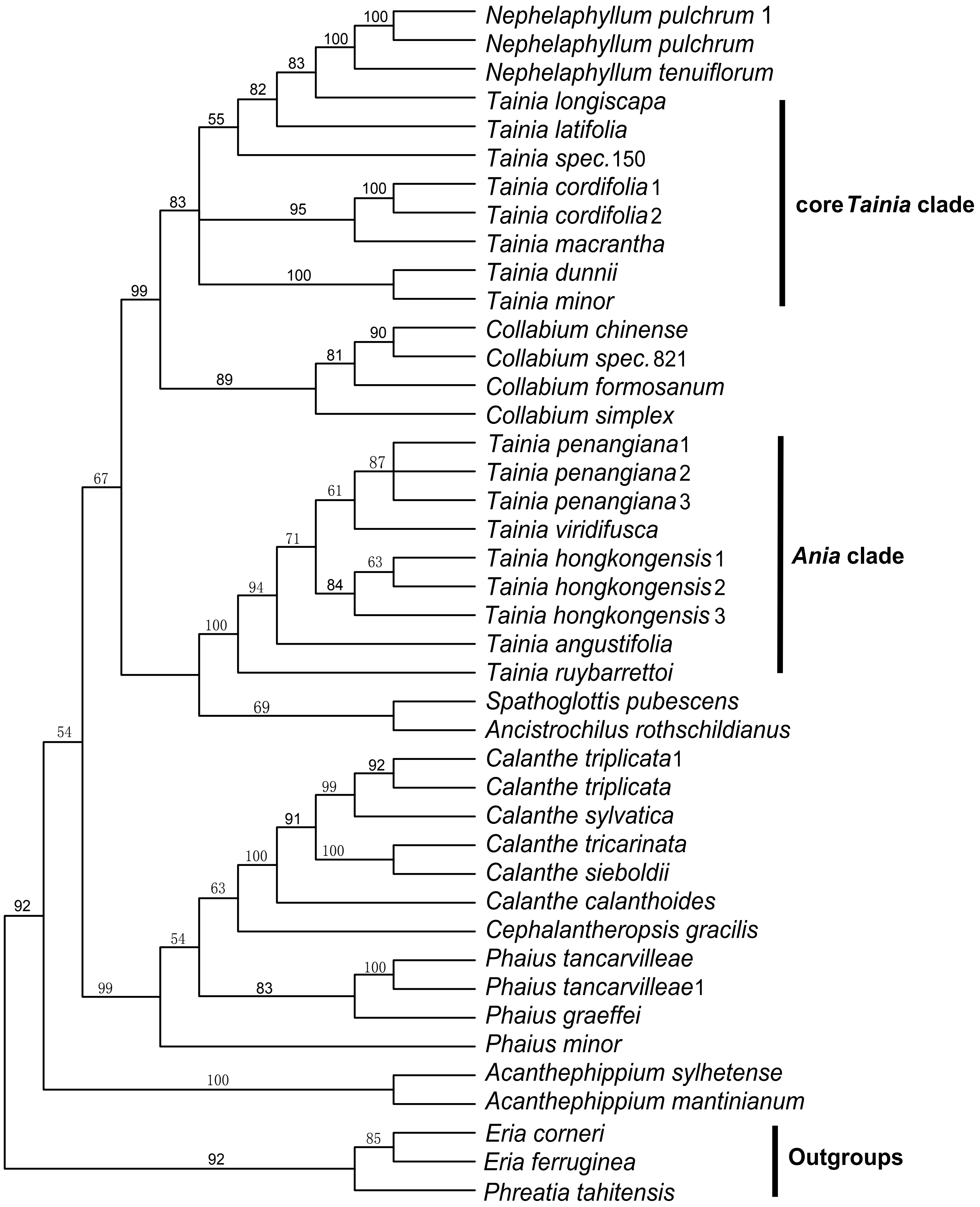

Fig. S1. Strict consensus tree of 3 equally parsimonious trees inferred using nr DNA ITS dataset to show the placement of *Ania* and the core *Tainia*. Bootstrap values are indicated above.
