# Supplementary material for: Re-Establishment of the Genus Ania Lindl. (Orchidaceae)
Source: PLoS One. 2014 Jul 21;9(7):e103129. doi: 10.1371/journal.pone.0103129 (PMC4105443; doi:10.1371/journal.pone.0103129)
Supplement: Figure S2 — Strict consensus tree of 675 equally parsimonious trees inferred using cp DNA trnL intron dataset. Bootstrap values are indicated above(>50%). (PDF) [file pone.0103129.s002.pdf]

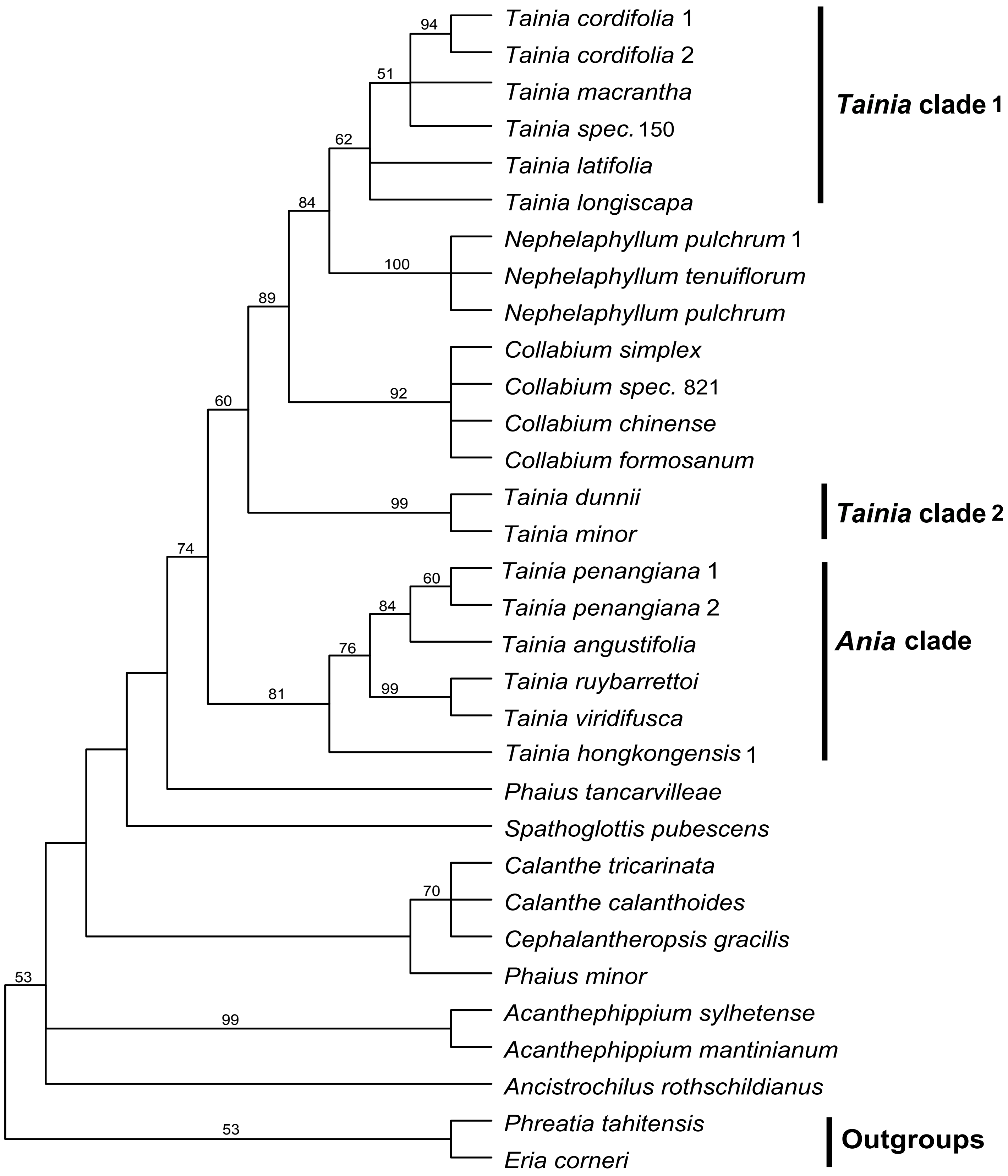

Fig. S2. Strict consensus tree of 675 equally parsimonious trees inferred using cp DNA *trnL* intron dataset to show the placement of *Ania* and *Tainia.* Bootstrap values are indicated above (> 50%).
