# Supplementary material for: Re-Establishment of the Genus Ania Lindl. (Orchidaceae)
Source: PLoS One. 2014 Jul 21;9(7):e103129. doi: 10.1371/journal.pone.0103129 (PMC4105443; doi:10.1371/journal.pone.0103129)
Supplement: Figure S3 — Maximum likelihood phylogeny of the combined datasets (ITS and trnL intron) with bootstrap values on the branches (>50%) to show the placement of Ania and the core Tainia . (PDF) [file pone.0103129.s003.pdf]

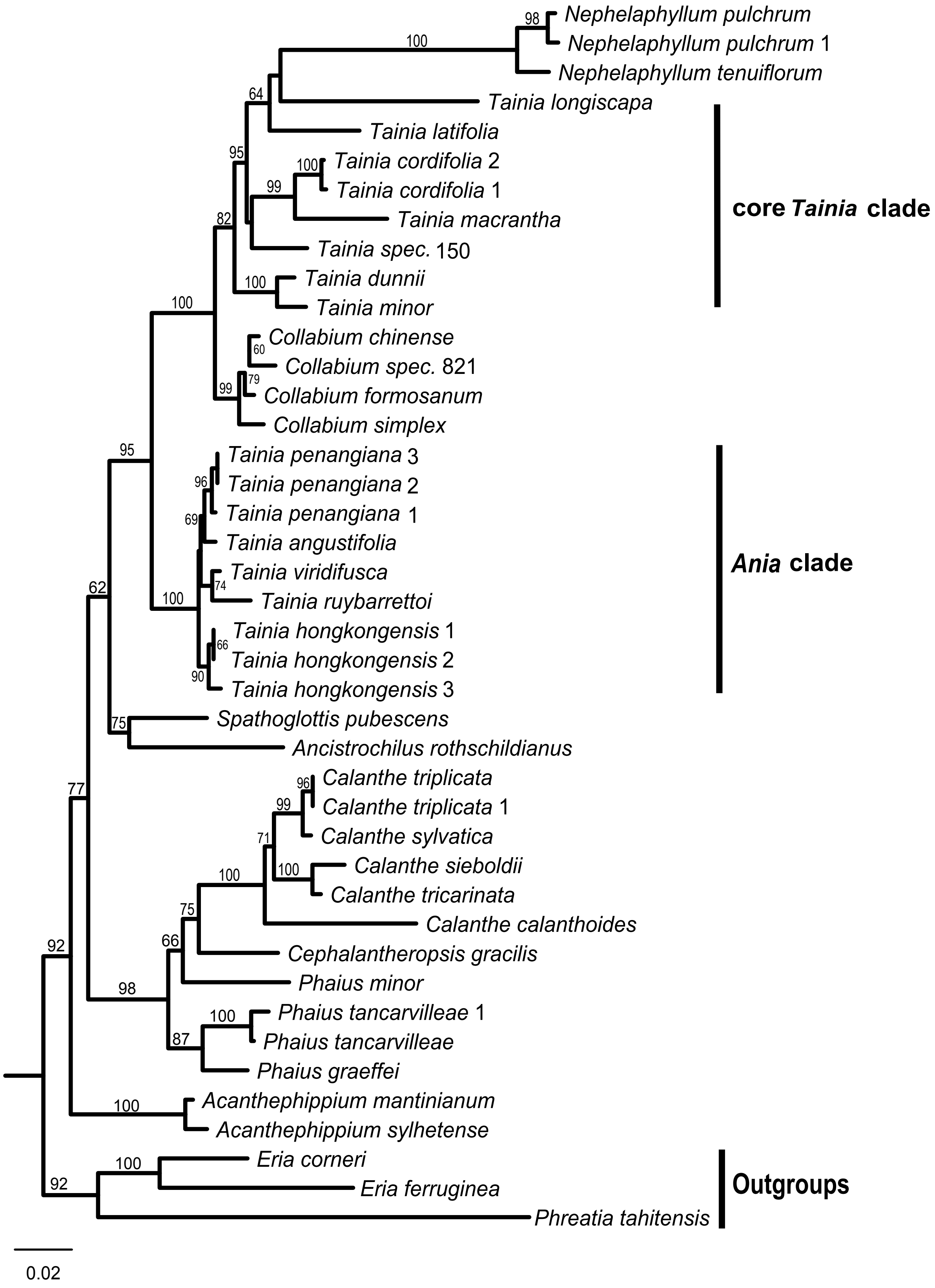

Fig.S3. Maximum likelihood phylogeny of the combined datasets (ITS and *trnL* intron) with bootstrap values on the branches (> 50%) to show the placement of *Ania* and the core *Tainia.*
