# Supplementary material for: Re-Establishment of the Genus Ania Lindl. (Orchidaceae)
Source: PLoS One. 2014 Jul 21;9(7):e103129. doi: 10.1371/journal.pone.0103129 (PMC4105443; doi:10.1371/journal.pone.0103129)
Supplement: Table S1 — Voucher information and GenBank accession numbers of the sampled taxa in the molecular analysis. Sequences generated in this study are marked with an asterisk (*). (DOC) [file pone.0103129.s004.doc]

**Table S1.** **Voucher information and GenBank accession numbers of the sampled taxa in the molecular analysis. Sequences generated in this study are marked with an asterisk (*).**

| Taxon, locality, collector and number and herbarium for voucher specimen, GenBank accession numbers: ITS, *trnL* intron ( *trnL* intron information follows nrITS information). The number after the second hyphen in a voucher, e.g., “-1” indicates the individual in a sampled population. Markers noted # are sequences not available. |
| --- |
| ***Acanthephippium mantinianum*** L.Lind. & Cogn., AF521081, AF519966; ******Acanthephippium sylhetense*** Lindl., Yunnan, China, *L. Li 124* (IBSC), KM025144, KM025171; ***Ancistrochilus rothscildianus*** O'Brien, AF521061, AF519928-9; ******Ania angustifolia*** Lindl., Yunnan, China, *L. Li 064* (IBSC), KM025145, KM025172; ******Ania hongkongensis*** (Rolfe) T. Tang & F.T.Wang-1, Guangdong, China, *L. Li 099* (IBSC), KM025146, KM025173; ******Ania hongkongensis*** (Rolfe) T. Tang & F.T.Wang-2, Hainan, China, *L. Li 140* (IBSC), KM025147, *#*; ******Ania hongkongensis*** (Rolfe) T. Tang & F.T.Wang-3, Guangdong, China, *L. Li 101* (IBSC), KM025148, *#*; ******Ania penangiana*** (Hook. f.) Summerh. -1, Yunnan, China, *L. Li 112* (IBSC), KM025149, KM025174; ******Ania penangiana*** (Hook. f.) Summerh. -2, Hainan, China, *L. Li 113* (IBSC), KM025150, KM025175; ******Ania penangiana*** (Hook. f.) Summerh. -3, Guangdong, China, *L. Li 128* (IBSC), KM025151, *#*; ******Ania ruybarrettoi*** S.Y. Hu & Barretto, Hainan, China, *L. Li 097* (IBSC), KM025152, KM025176; ******Ania viridifusca*** (Hook.) Tang & F.T.Wang ex Summerh., Yunnan, China, *L. Li 069* (IBSC), KM025153, KM025177; ***Calanthe calanthoides*** (A. Rich. & Galeotti) Hamer & Garay, AF521063, AF519941-2; ***Calanthe sieboldii*** Decne. ex Regel, AY882613, #; ***Calanthe sylvatica*** (Thouars) Lindl., AB222034, #; ***Calanthe tricarinata*** Lindl., AY882603, AF519940; ***Calanthe triplicata*** (Willemet) Ames-1, AY882614, #; ****Calanthe triplicata*** (Willemet) Ames, Hainan, China, *L. Li 126* (IBSC), KM025154, *#*; ****Cephalantheropsis gracilis*** (Lindl.) S.Y. Hu, Hainan, China, *L. Li 048* (IBSC), KM025155, KM025178; ****Collabium chinense*** (Rolfe) Tang & F.T. Wang, Guangdong, China, *L. Li 121* (IBSC), KM025156, KM025179; ******Collabium formosanum*** Hayata, *L. Li 122* (IBSC), KM025157, KM025180; ***Collabium simplex*** Rchb. f., EF670387, EF67043; ***Collabium sp.***, AF521052, AF519910-1; ****Eria corneri*** Rchb. f., Guangdong, China, *L. Li 146* (IBSC), KM025158, KM025181; ***Eria ferruginea*** Lindl., AF521071, #; ****Nephelaphyllum tenuiflorum*** Bl., Hainan, China, *L. Li 047* (IBSC), KM025159, KM025182; ****Nephelaphyllum pulchrum*** Bl., Hainan, China, *L. Li 119* (IBSC), KM025160, KM025183; ***Nephelaphyllum pulchrum*** Bl.-1, AF521070, AF519952-3; ***Phaius graeffei*** Rchb. f., AB222033, #; ***Phaius minor*** Bl., AF521051, AF519907; ****Phaius tancarvilleae*** (L'Hér.) Bl., Hainan, China, *L. Li 125* (IBSC), KM025161, KM025184; ***Phaius tancarvilleae*** (L'Hér.) Bl.-1, AB222032, #; ***Phreatia tahitensis*** Lindl., AF521065, AF519944; ****Spathoglottis pubescens*** Lindl., Guangdong, China, *L. Li 055* (IBSC), KM025162, KM025185; ****Tainia cordifolia*** Hook. f. -1, Guangdong, China, *L. Li 120* (IBSC), KM025163, KM025186; ****Tainia cordifolia*** Hook. f.-2, Taiwan, China, *L. Li 142* (IBSC), KM025164, KM025187; ****Tainia dunnii*** Rolfe, Guangdong, China, *L. Li 111* (IBSC), KM025165, KM025188; ****Tainia latifolia*** (Lindl.) Rchb. f., Yunnan, China, *L. Li 060* (IBSC), KM025166, KM025189; ****Tainia longiscapa*** (Seidenf. ex H. Turner) J.J. Wood & A.L. Lamb, Hainan, China, *A Q. Hu & L. Li 057* (IBSC), KM025167, KM025190; ****Tainia******macrantha*** Hook. f., Guangdong, China, *L. Li 093* (IBSC), KM025168, KM025191; ****Tainia minor*** Hook. f., Yunnan, China, *L. Li 143* (IBSC), KM025169, KM025192; ****Tainia sp*.,** Hainan, China, *L. Li 150* (IBSC), KM025170, KM025193. |
