# Supplementary material for: Re-Establishment of the Genus Ania Lindl. (Orchidaceae)
Source: PLoS One. 2014 Jul 21;9(7):e103129. doi: 10.1371/journal.pone.0103129 (PMC4105443; doi:10.1371/journal.pone.0103129)
Supplement: Table S2 — Morphological characters included in the cladistic analysis (1–14, vegetative characters; 15–50, reproductive characters). (DOC) [file pone.0103129.s005.doc]

**Table S2. Morphological characters included in the cladistic analysis (1--14, vegetative characters; 15--50, reproductive characters).**

| **1. Habitat types:** (0) terrestrials; (1) epiphytes, lithophytes, rarely terrestrials. **2. Rhizome diameter:** (0) thin, no more than 3 mm; (1) thick, 4--6 mm. **3.** **Pseudobulb shape:** (0) slender, petiole-like, cylindrical; (1) dilated at base, cylindric-conical; (2) swollen ovoid to conical; (3) gourd-shaped. **4.** **Pseudobulb width at widest point (fresh):** (0) very thin, 1.5--4 mm; (1) thin, 4--15 mm; (2) thick, 20--80 mm. **5.** **Pseudobulb surface (mature):** (0) smooth, not angled; (1) conspicuously angled or with several slight ridges. **6.** **Leaf number:** (0) solitary; (1) two or more. **7. Leaf texture:** (0) thin, paper; (1) slightly thickened and fleshy; (2) leather. **8. Leaf vernation:** (0) conduplicate; (1) not distinctly conduplicate or plicate; (2) plicate. **9. Leaf shape:** (0) cordate or ovate; (1) oblong, linear-lanceolate or elliptical. **10.** **Leaf base:** (0) cordate to rounded; (1) cuneate to acute. **11.** **Leaf articulation:** (0) absent; (1) present. **12. Leaf adaxial:** (0) marbled pattern absent; (1) grey-green or purplish marbled pattern present. **13.** **Leaf abaxial:** (0) green, not purplish; (1) purplish or dull purplish. **14.** **Petiole:** (0) sessile or sub-sessile; (1) short, indistinct, between 0.3--1.5cm; (2) between 2--4.5 cm, not more than 5 cm; (3) long and distinct, usually longer than 5 cm, up to 5--38 cm. **15. Inflorescence position:** (0) terminal; (1) lateral or axillary. **16.** **Inflorescence pubescence:** (0) absent; (1) present. **17. Stellate hairs of inflorescences:** (0) absent; (1) present. **18. Peduncle length:** (0) not more than 3 cm; (1) 10--20 cm long, not more than 23 cm; (2) up to 23--156 cm long. **19.** **Flower number:** (0) single; (1) two or more than two flowers. **20.** **Flower position:** (0) non-resupinate; (1) resupinate. **21. Flower bilateral symmetry:** (0) present; (1) absent. **22. Tepal shape:** (0) not caudate; (1) caudate. **23. Abaxial hairs of sepals:** (0) absent; (1) present. **24. Petal length (average):** (0) usually 7--13 mm, no more than 13 mm; (1) longer than 13 mm, usually 13.5--32 mm; (2) longer than 32 mm, usually 33--45 mm. **25.** **Petal width (average):** (0) narrower than 2.5 mm; (1) usually 3--5 mm; (2) usually broader than 5 mm, sometimes up to 10 mm. **26.** **Width ratio of lateral** **sepals to petals:** (0) less than 1; (1) greater than or equal to 1. **27.** **Lateral sepal shape:** (0) Lateral sepal widest near middle, elliptic-ovate to oblong-ovate, not or only slightly widened at base, not triangular; (1) distinctly widened below the middle, widest at base, tapered to apex, more or less triangular to ovate-triangular, sometimes caudate-acuminate; (2) distinctly widened below the middle, widest at base, broadly triangular-ovate. **28**. **Lateral sepals inserted on column-foot:** (0) absent; (1) narrowly inserted; (2) broadly inserted. **29. Lateral sepals basally connate:** (0) absent; (1) present. **30.** **Lip base:** (0) not spurred or somewhat saccate; (1) shallowly saccate or shortly spurred, 2--6 mm long; (2) prominently saccate or long spurred, slender and tubular, more than 1.5 cm long. **31. Lip disk:** (0) keels absent or with low, linear ridges; (1) keels present and conspicuous; (2) keels absent, covered by long hairs or plate-like appendage. **32. Lip claw:** (0) absent; (1) present, claw with straight margins; (2) present, claw with pleated margins. **33. Lip lobes:** (0) absent; (1) present. **34.** **Lip length:** (0) not more than 5 mm; (1) usually 6--15 mm; (2) longer than 15 mm, up to 16--35 mm. **35. Lip motility:** (0) absent; (1) present. **36.** **Mentum:** (0) absent or indistinct; (1) conic, not more than 2 mm; (2) spurlike, cylindric, 4--6 mm. **37. Column length:** (0) short, 2.5--6 mm; (1) usually 6.5--11 mm; (2) up to 15 mm. **38.** **Column orientation:** (0) porrect or slightly arcuate; (1) conspicuously arcuate. **39. Top margins of column:** (0) entire or inconspicuously denticulate; (1) conspicuously denticulate to erose. **40.** **Column wing:** (0) present only at the apex or conspicuously broader in the distal part; (1) present over total length or wings with proximal and distal parts subequal. **41.** **Column with decurved toothlike projections on each side:** (0) absent; (1) present. **42.** **Fleshy keels of column base:** (0) absent; (1) present. **43. Column-foot spur:** (0) absent; (1) present. **44. Column-foot:** (0) absent or indistinct, not more than 1.5 mm; (1) 2--7 mm; (2) long and prominent, 10--17 mm. **45. Anther crest:** (0) absent; (1) present. **46.** **Pollinium number:** (0) 2; (1) 8. **47.** **Caudicles:** (0) absent; (1) present. **48.** **Pollinium shape:** (0) not laterally compressed; (1) laterally compressed. **49. Pollinium type:** (0) homomorphic or subequal; (1) heteromorphic or distinctly unequal. **50.** **Pollen** **exine ornamentation:** (0) seam sculpturing absent or indistinct; (1) seam sculpturing distinct. |
| --- |
