# Supplementary material for: Re-Establishment of the Genus Ania Lindl. (Orchidaceae)
Source: PLoS One. 2014 Jul 21;9(7):e103129. doi: 10.1371/journal.pone.0103129 (PMC4105443; doi:10.1371/journal.pone.0103129)
Supplement: Table S3 — Details of accessions included in the morphological study. (DOC) [file pone.0103129.s006.doc]

**Table S3. Details of accessions included in the morphological study.**

| Taxon, collection locality, collector and number (herbarium) for all specimens of that species. |
| --- |
| ***Ania angustifolia*** Lindl., **Myanmar:** Tenasserim, Tavoy, *Gomez s.n.* (K, BM, E), locality unknown, *Kingdon-ward 22717* (BM). **Thailand:** Chiengmai, Doi Sutep, *Kerr 195* (K), Doi Chieng Dao, *Put 297* (K), *Maxwell 87-1002* (L), *Maxwell 88-1082* (L), Mai Muang Nao Arboretum, S*ankamethawee 284* (L), Lampoon, Doi Kuhn Dahn National Park, *Maxwell 93-1074* (L). **Vietnam:** Annam, *Evrárd 2387* (P), Lam Dong, Dalat, *Tixier s.n.* (P), *Evrárd 1457* (P). **China:** Yunnan, *Li* *80-1114* (KUN), *Li 064* (IBSC), *Li 075* (IBSC), *Tsi 91-433* (K, PE, MO), *Tsi 92-378* (MO), *Wang 75690* (PE, KUN), *Wang 75882* (PE, KUN). ***Ania elmeri*** (Ames) A.D. Hawkes ex Senghas, **Philippines:** Luzon, *Vanoverbergh 102* (L, MO, P), *Vanoverbergh* 2*434* (K, BM), *Vanoverbergh* *4052* (US), Manila, *Loher 541* (K), *Loher 14662* (US). ***Ania hongkongensis*** (Rolfe) T. Tang & F.T.Wang, **China:** Hong Kong, locality unknown, *Chun* *42573* (IBSC), *Wright* *522* (AMES, K, NY, P), Landau Island, *Xu 023063* (SZG), Happy valley, *Bodinier 1100* (E, P), *Ford 11* (K), Mt. Parker, *Wilford 384* (K), Chung Chi College, *Hu 9253* (K, US). Guangdong, *How 70182* (IBK, PE), *Ding & Shi 286* (IBSC, HITBC, MO), *Shi 11227* (IBSC), *Shi 12770* (IBSC), *Levine s.n.* (PE), *Li 101*(IBSC), *Li 128* (IBSC), *Li 129* (IBSC), *Li 130* (IBSC), *Chun 42576* (IBSC), *Chen & Zhang 511* (IBSC), *Chun 42576* (KUN), *Li 099* (IBSC), *Li 114* (IBSC). Shenzhen, *Zhang, Li et al. 038*1(SZG), *Zhang, Li et al. 1631* (SZG), Zhang, *Li et al. 3171* (SZG), *Zhang, Chun et al. 3864* (SZG), *Chun, Zeng et al. 5752* (SZG), *Shenzhen Exploration Team 013452* (SZG). Hainan, *Li 140* (IBSC), *Li 144*(IBSC). **Vietnam:** Quang Tri, *Eberhardt 1975* (P). ***Ania penangiana*** (Hook. f.) Summerh., **Malaysia:** Penang, *Maingay 1642* (K, L), Government Hill, *Curtis s.n.* (K), Sabah, Tuaran, *Collenette 73* (BM), Mt. Kinabalu, *Gibbs 3958* (K, BM), *J. &* *M.S.Clemens 30125* (E, L), Tambunan, *Lamb AL 321/85* (K), near Rarau, *Collenette 73* (BM). **Indonesia:** Java, *Comber 1082* (K), Sumatra, *Van Steenis 8960* (L). **China:** Yunnan, *Ye & Li 074*(IBSC), *Ye & Li 112*(IBSC). Guangdong, *Chun 8887* (PE), Chun 8888 (PE). Hainan, *Hainan Western Team 93* (PE), Bawangling, *Li et al. 5072*(IBSC), *How & Chun 70182* (IBSC), *Liang 65114* (IBSC), *Li* 073 (IBSC), *Li 127* (IBSC). **India:** Bengal, Western Duars, *Haines 2839* (K), Orissa, Jenabil, Similipal forest, *Sarat Misra 1080* (K). **Sikkim Himalaya:** *Pantling 204* (AMES, BM, K, P). **Thailand:** Chiengmai, Doi Suthep, *Kerr 214* (K), *Kerr 364* (K), *Maxwell 90-140* (L), Sangka, *Kerr 0128* (K), Dan Chumphon, *Kerr 0758* (K), Lampoon: Doi Kuhn Dahn National Park, *Maxwell 94-157* (L), Ranong, *Kerr 0689* (K). **Vietnam:** Lam Dong, Dalat, Manline, Tixier 8/59 (P), Baria, Mt. Dinh, *Pierre 6675* (P). ***Ania ponggolensis*** A. Lamb, **Malaysia:** Sabah, Batu Ponggol, *Lamb 204/84* (K, L), *Chan s.n.* (K). ***Ania ruybarrettoi*** S.Y. Hu & Barretto, **China:** Hong Kong, Mt. Tai Mo Shan, *Hu 13098A* (K, PE). Guangxi, Pingnan, *Huang 39145* (IBSC, PE). Hainan, *Li 059* (IBSC), *Li 097* (IBSC). ***Ania viridifusca*** (Hook.) Tang & F.T.Wang ex Summerh., **India:** Assam, cult. in Kew, *Simon s.n.* (K), Chin Hills, *Kingdon Ward 21737* (BM), Manipur, *Watt 6293* (K), Nagaland, *Kingdon Ward 11209* (CAL). **Sikkim Himalaya:** *Pantling 106* (BM). **Vietnam:** Tonkin, Bac Kan, *Eberhardt 4673* (P), Thai Nguyen, Cho Chu, *Eberhardt 3984* (P), Yen Bai, *Rives s.n.*(P).**Thailand:** Chiengmai, Doi Suthep, *Kerr 226* (K), *Kerr 226A* (K), *Kerr s.n.*(K), Meo Village, *Maxwell 91-148* (L), Mt. Doi Inthanond, *Garrett 632* (K), Lampoon, Doi Kuhn Dahn National Park, *Maxwell 94-171* (L), Udawn, Loei, Mt. Phu Luang, *Hennipman 3578* (L). **Myanmar:** Amherst, *Lace 5622* (K, CAL), Tenasserim, Moulmein, *Parish 73* (K), *Parish 244* (K), Karan, Mulayit Taung, Amherst, *Parkinson 5113* (K), Kachin, Htawjaw, Naung Chaung Valley, *Kingdon Ward 1721* (E), Laktang, *Kingdon Ward 3229* (E), The Triangle, *Kingdon Ward 20615* (BM). **China:** Yunnan, *Exploration Team 23824* (HITBC), *George Forrest 17652* (E), *Henry 11813* (K), *Li 069*(IBSC), *Li* 115(IBSC), *Li 116*(IBSC), *Li 117*(IBSC), *Qian 3573* (HITBC), *Wang 74418* (IBSC). ***Chrysoglossum assamicum*** Hook.f., **India:** Assam, *Griffith 1233/1322* (K). **China:** Guangxi, *Huang 39101* (PE), *Xin 8090* (PE). Hainan, *Li 105* (IBSC). Xizang, *Li & Cheng 02702* (PE). ***Chrysoglossum ornatum*** Bl., **Indonesia:** Java, Mt. Salak, *Blume 295* (K, L), Mt. Pajung, *Wirawan 290* (L), Bogor, *Holttum s.n.* (K), *Sun 2262* (PE). **Thailand:** Khao Keo, *Kerr 0535* (K), Ranong, *Kerr 0690* (K), Doi Sutep, *Kerr 352* (K). **China:** Yunnan, *Liu 0114* (PE), *Qiu 52687* (PE), *Tsi 194* (PE), *Wang 74418* (PE), *Yu 18197* (PE, KUN), *Zhang 185* (PE). **Taiwan:** Kaohsiung, *Lin s.n.* (TAI). **Sikkim Himalaya:** Rishap, *Clarke 12314* (K). **Bhutan:** Lachong Valley, *Pantling 83* (P). ***Collabium chinense*** (Rolfe) Tang & F.T. Wang, **China:** Fujian, *Xiamen University Acquisition Team 1240* (KUN, PE), *Ye 1677* (PE, IBK), *Zhuang 8305057* (PE). Guangdong, *Hance 17733* (BM), *Hau 73447* (PE), *Chun 7075* (IBK), *Li 121* (IBSC). Guangxi, *Liu 28440* (IBK). Hainan, *Hau 73447* (IBK), *Liu 28005* (IBK). Xizang, *Tibetan Team 74-437*9 (KUN). Yunnan, *Mao 02838* (PE, IBK). **Taiwan:** Taipei, *Zhang 263812* (TAI), Nantou, *Zhang 271852* (TAI). **Thailand:** Kao Nawng, *Robinson s.n.* (K), *Kerr 0445* (K). **Vietnam:** Mt. Bavi, *Balansa 1998* (P). ***Collabium delavayi*** (Gagnep.) Seidenf., **China:** Yunnan, *Delavay s.n.* (P). ***Collabium formosanum*** Hayata, **China:** Fujian, *Longxi Moutain Expedition Team 2355* (PE), Guangdong, *To & Tsang 12402* (PE, IBSC), *Li 122* (IBSC). Guangxi, *Guangfu Forests Investigation Team 571* (MO), *Chun 14100* (IBK), *Chun 14120* (KUN), *Chun 14439* (IBK, PE, KUN), *Liuzhou Team 2819* (IBK), *LÜ 2819* (IBK, PE, KUN), Guizhou, *Peking Youth Team 0352* (PE), Hunan, *Zhao 764655* (PE). Sichuan, *China-USA Scientific Exploration Team s.n.* (PE). Yunan, *Feng 11913* (KUN), *Mao 02536* (PE, KUN), *Sui 001146* (PE), *Sui 002199* (PE), *Wu 8048* (KUN). **Taiwan:** Taipei, *Suzuki-Tokio 16798* (TAI), Yilan, *Huang et al. 5201* (TAI), Xinzhu, *Xu 522* (TAIF). **Vietnam:** Tonkin,*Pételot 5148* (P), *Pételot 5165* (P), *Pételot 5880* (P). ***Diglyphosa latifolia*** Bl., **Indonesia:** Java, *Blume s.n.* (L), *Zollinger 680 Z* (P). **Philippine:** *Elmer 11815* (AMES). **China:** Yunnan, *Tsai 60311* (PE, KUN), *Anonymous 8276* (PE). **India:** Chel Valley, *Pantling 315* (P). ***Eria corneri*** Rchb. f., **Vietnam:** Annam, *Poilane 24142* (P). Kontum, *Averyanov 1938* (P). Tonkin, *Bon 3062* (P). **China**: Guangdong, *Li 146* (IBSC), *Liu* *23942* (IBK, IBSC, KUN, PE), *Tsang 26747* (IBSC, P). Guangxi, *Wei & Liu M0078* (IBK), *Wei & Liu M0173* (IBK). Yunnan, *Tsi 192* (PE), *Wang 79337* (KUN), *Wang 86145* (KUN), *Wang 87037* (KUN), *Zhang 141* (PE). Hainan, *Liang 62535* (IBK, PE), *Xia 5808* (PE). Hong Kong, *Chan 1137* (P), *Furet 259* (P), *Furet 906* (P). ***Eria ferruginea*** Lindl., **India:** Assam, Jharam, *Prain 102* (P), locality unknown, *Loddiges s.n.* (K). ***Hancockia uniflora*** Rolfe, **Vietnam:** Tonkin, *Pételot 3561* (P, US). **China:** Yunnan, *Henry 11112* (K, NY), *Dawei Mountain Expedition Team D2125* (KUN), *Mao 02532* (PE, KUN), *Wang 85585* (PE, KUN). ***Nephelaphyllum tenuiflorum*** Bl., **Indonesia:** Java, *Blumn s.n.* (NY), *Lecomte 223* (P), *Lindley s.n.* (P), *Zollinger 1261* (P), *Anonymous 8457* (PE). **Thailand:** Nakhon Nayok, *King 5551* (US). **China**: Hong Kong, *Ford 4888* (P). Hainan, *Jin 9038* (PE), *Liu 27344* (IBK), *Li 047* (IBSC). **Vietnam:** Annam, *Poilane 18228* (P). Kontum, *Averyanov vh881* (P). Tonkin, *Balansa 2001* (P). **India:** East Bengal, *Griffith 5371* (P), Mt. Khasia, *Hooker s.n.* (P). ***Nephelaphyllum pulchrum*** Bl., **Malaysia:** Sarawak, *Yong 156* (K). **Indonesia:** Java, *Zollinger 2600* (P). Sumatra, *Bünnemeijer 3945* (P). **Thailand:** Bachaw, Pattani, *Kerr 035* (K). Bangkok, *Kerr s.n.* (K). Nam Nao, Petchaboon, Smitinand (P), **India:** East Bengal, *Griffith 5370* (K). **China**: Hainan, *Song 121*(PE), *Song 134* (PE), *Li 119* (IBSC). ***Tainia bicornis*** Rchb. f., **Sri Lanka:** *Clowes s.n.* (K). **India:** Kerala, *Mohanan 59301*(CAL), *Nair 69830* (CAL), *Nair 65491* (CAL), Madras (Chennai), *Manantoddy 1284* (K), Mysore, *Saldanha 15953* (E, MO, US), *Saldanha 16181* (US), Tanil Nadu, *Chandralose 65897* (CAL), *Gopalan 81415*(CAL), *Srinivasan 68039* (CAL), *Srinivasan 70362* (CAL). Naterikal forest, *Joseph 15846* (CAL). ***Tainia cordifolia*** Hook. f., **China:** Guangdong, *Tso 22753*(IBSC), *Liang 60691* (IBSC, KUN), *Chun 42840* (IBSC), Dapu *Exploration Team 452* (IBSC), *Tsang 21097* (PE, K), *Li 120* (IBSC). Guangxi, *Qin 700247* (IBSC), *Tsang 22260* (IBSC). Fujian, *Ye 1493* (PE), *Xiamen University Acquisition Team 1338* (PE), *Lin 391* (IBSC, PE), *Wang 1212* (PE). Yunnan, *Wang & Liu 86007* (PE). **Taiwan:** Keelung, *Ford 56* (K), Taipei, Feitsui Reservoir, *Lin & Shen s.n.* (TAI), Wulai, *Chen 97* (HAST), *Leu 706* (HAST), Chen 76 (HAST), *Yamamot s.n.*(TAI), Daren, *Kao 9642* (TAI), locality unknown, *Zhang & Li 142* (IBSC). **India:** Mt. Kushaku, *Faurie 539* (P). ***Tainia crassa*** (H. Turner) J.J. Wood & A.L. Lamb, **Malaysia:** cult. in Bot. Gard. Edinburgh, *Burtt & Woods B1609* (E). ***Tainia dunnii*** Rolfe, **China:** Guangdong, *Teng 5062* (IBSC), *Tso 22754* (IBSC), *Chun 42727* (IBSC, IBK), *Kao 52606* (IBSC, PE), *Yue 71 167* (IBSC), *Guo* 80305 (IBSC), *Tso 42727* (PE), *Qiu 60857* (IBSC), *To & Tsang 12246* (IBSC, AMES), *Deng 1322* (IBSC, MO), *Nanling Exploration Team 2300* (IBSC), *Ko 51493* (IBSC), *Sin 9445* (IBSC), *Tsang 20457* (IBSC, PE, K, US), *Li 111* (IBSC), *Li 123* (IBSC), *Kao50395* (IBSC), locality unknown, *Li 200661* (PE, IBK, MO), *Li 118* (IBSC), *Dalziel s.n.*(E). Guangxi, *Sin 21271* (IBSC), *Sin 23104* (IBSC), *Dayaoshan Exploration Team 10754* (IBSC, IBK); *Chun 14393* (IBSC, IBK), *LÜ 2850* (IBSC, IBK, PE, KUN, HITBC), Liuzhou *Exploration Team 2850* (IBK), *Guangfu Forests Investigation Team 525* (IBSC, IBK). Fujian, *Dunn 3542* (K), *He 2030* (PE), *Li 204* (IBSC), *Ye 02071* (PE, MO), *Huang 190303* (IBSC), *Ye 1480* (PE), *Lin 432* (PE), *Lin 1332* (PE), *He 0480* (PE), *Li* *10907* (PE). Taiwan, Hualian, Mt. Qingshuishan, *Shimizu & Kao s.n.*(TAI), Taibawang, *Ying 3511B* (NTUF), Pingdong, *Matuda 596* (TAI), *Wang 16094* (HAST), *Chen 502* (HAST), *Wu 1168* (HAST), Taipei, *Shimada s.n.* (TAIF), *Peng 10131* (HAST), *Yushunkudo s.n.*(TAI), Wulai, *Leu 1420* (HAST), *Liao 1124* (HAST), Su *8604* (HAST), *Su 8797* (HAST), *Su 1503* (NTUF), *Suzuki-Tokio 16481* (TAI), *Suzuki-Tokio 18561* (TAI), Beitou, *Lin s.n.*(TAI), *Chang s.n.*(TAI), Miaoli, Taian, *Liu 130* (HAST), *Liu 133* (HAST), Nanzhuang, *Fukuyama 3393* (TAI), Taidong, Donghe, Y. C. *Kao 770* (HAST), Chenggong, *Su 8246* (HAST), Gaoxiong, Xinzhu, *Su 8230* (HAST), *Su 8928* (HAST), *Su 8929* (HAST), *Su 8935* (HAST). Hunan, *Huang 112174* (IBSC), *Yang 408* (IBSC), *Zhao* *763396* (PE), *Zhao* 764493 (PE), *Zhao* *764498* (PE), *Zhao* *764514* (PE), *Luo* *2317* (PE), *Luo 2061* (PE). Jiangxi, *Lai 4195* (IBSC, IBK, KUN), *Nie et al. 08249* (IBSC, IBK, KUN), *236 Task Group548* (PE), *236 Task Group*797 (PE), *Wan & Yu s.n.* (PE), *Jiangxi Team 1035* (PE), *Tan 98149* (SZG), *Ye 7200* (MO), *Ye* *7316* (MO). Zhejiang, *He 22895* (PE), *He 28439* (PE), *Zuo et al. 23821* (PE), *Zhenjiang Plant Resource Exploration Team 25736* (PE), *Zhenjiang Plant Resource Exploration Team 26858* (PE), *Zhenjiang Plant Resource Exploration Team 26197* (PE). Sichuan, *236 Sichuan Task Group 2002* (PE), 2035 (PE), *Lang, Kao et al. 016* (PE), *Lang, Kao et al. 050* (PE), *Guan, Wang et al.* *1269* (PE), *Guan, Wang et al.* *1282* (PE), *Guan 8983* (PE), *Yao 2248* (PE). Guizhou, *Deng 90530* (IBSC), *Deng 90630* (IBSC). ***Tainia latifolia*** (Lindl.) Rchb. f., **Myanmar:** Tenasserim, *Parish 253* (K), *Griffith 5288* (P), *Namtuseek*, *Griffith s.n.*(K), Kachin, Kachin Hills, *Chin 5761* (K), Tavoy, *Russell 50* (CAL). **India:** Assam, *Griffith s.n.* (K), *Prain 855* (CAL), *Simon s.n.*(K); Cachar, Katakhal Forest, *Mann s.n.*(K, E), Bengal, Khasia Hills, *Hooker & Thomson 217* (K), *Clarke 42949* (K), Lushai Hills, *Parry 126* (K), Makum Forest, *Brandis s.n.*(K), Similipal, *King & Pantl. SM 1080* (CAL), Slhet, *Bruce s.n.* (K). **Sikkim Himalaya:** *Pantling* *106* (BM, CAL, E, K, P). **China:** Yunnan, *The Sino-Soviet Joint Investigation of Yunnan 7653* (IBSC, KUN), *Zhu* *2248* (KUN), *The* Sino-Soviet Joint *Investigation of Yunnan 5419* (KUN), *Liu 0114* (KUN), *Li 060* (IBSC), *Li 102* (IBSC), *Li 132* (IBSC), Hu *& Li 080* (IBSC), *Henry 12078* (K, MO, US). **Thailand:** Phitsanulok: Khao Huey Khek, *Hansen & Smitinand 11201* (K, L). Chiang Mai: Doi Sutep, *Kerr 401* (K), Mt. Doi Pahompok, *Kerr 401* (K), near Chiengkhum, Hué Me Tla, *Kerr 295* (K, AMES), **Indonesia:** Java, *Sun 8562* (PE), *Sun s.n.* (PE), *Lobb 161* (BM), *Lobb s.n.*(K), *Danm 6790* (L), *Comber 1649* (K), Fjibeber, *Smith 744* (K, P), Sumatra, Labuah gunung, *Sarkat Danimihardja SD 2318* (L). **Malaysia:** Sabah, Ranau District, *Doinis Soibeh 725* (K). ***Tainia laxiflora*** Makino, **Japan:** Ohsumi, Yaku-shima, *Miyoshi Furuse 10785* (K), *Miyoshi Furuse 10821* (K), *Saito 1533* (BM, L), Ryukyu, *Miyoshi Furuse 5432* (K). ***Tainia longiscapa*** (Seidenf. ex H. Turner) J.J. Wood & A.L. Lamb, **Thailand:** locality unknown, *Seidenfaden & Smitinand GT 9684* (L). **China:** Yunnan, *Zhang* *156* (PE). Hainan, *Hu & Li 057* (IBSC). ***Tainia macrantha*** Hook. f., **China:** Guangdong, *Ford 153* (K), *Kao51680* (IBSC, KUN), *Huang 31142* (IBSC, PE, KUN), *Merrill 11011* (US), *Chun 41531* (IBSC), *Wang 31142* (IBK), *Li 093* (IBSC). Guangxi, *Chun* *14901* (IBSC, IBK, PE, KUN), *Chun* *14765* (IBSC, IBK, PE, KUN, HITBC), Hexian, *Chun et al.* *500292* (IBSC, IBK), *Longsheng Collection Team 50204* (IBK), *Sin 22349* (IBSC), *Sin 23659* (IBSC). Sichuan, Mt. Emeishan, *Lang, Gao et al. 023* (PE). **Vietnam:** Lao Cai: 16 km from Ta Phinh road, *Poilane 12798* (P).***Tainia maingayi*** Hook. f., **Malaysia:** Penang, *Maingay 1668* (K), *O’ Brien s.n.* (BM), Kedah, *Haniff 613* (K), Perak, *Curtis* *3290* (K), Selangor, *Carr 12025* (K). Sabah, *de Vogel 8455* (L), *Vermeulen & Duistermaat 911*(L). **Indonesia:** Mt. Palimasan, *Kostermans 13057* (L), Sumatra, *Latif s.n.* (L)**. *Tainia marmorata*** (J.J. Sm.) J.J. Wood & A.L. Lamb, **Indonesia:** Sulawesi, *Kjellberg 2939* (L). ***Tainia megalantha*** (T. Tang & F.T.Wang) ined., **Sikkim Himalaya:** *Pantling* *206* (K, AMES, P). ***Tainia minor*** Hook. f., **India:** Assam, *Prain 182* (CAL), Darjeeling, Mahalderam, *Clarke 35517* (BM, K), *Clarke 35517A* (K), *Hermans 2081*(K). **Sikkim Himalaya:** Mahalderam, *Clarke 35517* (K), *Kurz s.n. ex Sinchul* (BM), *Pantling 210* (K, BM, CAL, L), *King 701* (CAL). **China:** Yunnan, *The Sino-Soviet Joint Investigation of Yunnan 1172* (IBSC), *Feng 22314* (IBSC, KUN), *Feng 22315* (KUN), *The Sino-Soviet Joint Investigation of Yunnan 11112* (PE), *Jin & Li 143* (IBSC), *Tsi 133* (PE), *Jiang 03434* (YAF), *Tsi 93-04* (PE), *Tsi 145* (PE), *Tsi 228* (PE). **Myanmar:** Kachin, *Kingdon Ward 20938* (BM). ***Tainia obpandurata*** H.Turner, **Indonesia:** Sumatra, *Rahmat si Boeea 10356* (AMES, L), *Rahmat si Boeea 9710* (L), *Rahmat si Boeea 9804* (L), *Rahmat si Boeea 11412* (L), *De Wilde & de Wilde Duyfjes* *15720* (L), *De Wilde & de Wilde Duyfjes* *15863* (L). ***Tainia paucifolia*** J.J. Sm., **Malaysia:** Kelantan, *Henderson 19574* (K), *Henderson 24818* (K), Pahang, *Carr 275* (K), Perak, *Scortechini 759b* (K), Sabah, *Lamb T18* (K), *Lamb 1267/90* (L), *Nooteboom 1431* (L), Sarawak, *Brooke 10195* (L), *Carlo 872* (L). **Indonesia:** Java, *Comber 1259* (K), *Bakhuizen van den Brink 309* (L), *Docters van Leeuwen s.n.* (L), *Wirawan 290* (K, L), Sumatra, *Kostermans 22078* (L), Nakhon Si Thammarat, *van Beusekom & Phengkhlai 794* (E, K, L, P), *van Beusekom & Phengkhlai 875* (L). **Thailand:** *Kerr 535* (K), *Kerr 596* (K). *Cult in Hort. Bo. ex Tjianpea 145* (K). ***Tainia papuana*** J.J. Sm., **Papua New Guinea:** Morobe,*Apo* *& Umbas for Dockrill 34017*(K), *J. & M.S.Clemens 1268*(L), *J. & M.S.Clemens 1633*(L), *Millar NGF 9751* (K, L), *Millar NGF 13882*(K, L), *Millar NGF 18886*(E, K, L), *Millar NGF 23287* (L), *Millar NGF 22953*(L), *Woods 1075* (E), *Hartley 10998* (K, L), East Sepik, *Reeve 1148* (K), Lipan Pass, *Reeve 1150* (E, K, L), *Reeve 1268* (L), Keborr Valley, *P. van Royen 4955* (K, L), Markham, *P. van Royen NGF 20123* (L), *Stevens & Martin 54758* (L). **Pacific:** Solomon Islands, *Blaxell 3333* (L), *Brass 3235* (L). *Cult. in Hort. E. 6387* (E). ***Tainia*** [***purpureifolia***](http://plants.jstor.org/search?plantName=Tainia purpureifolia) [Carr](http://plants.jstor.org/search?personName=Carr), **Malaysia:** Sabah, Mt. Kinabalu, *Carr 3150* (AMES, K, SING), **Brunei:** Temburong, *De Vogel 9488* (L), *Coode 7477* (K). ***Tainia scapigera*** (Hook. f.) J.J. Sm., **Malaysia:** Kinabalu ridges, *Clemens s.n.* (BM), *Gibbs 2884* (BM), Sabah, *Lamb 197/84* (K), *Ridley s.n.* (K), *Surat AL 1363/91* (K). ***Tainia speciosa*** Bl., **Indonesia:** Java, *Blume s.n.* (L), *Backer 14173* (L), *Comber 1662* (K), *Lobb 159* (K, BM). **Malaysia:** Sabah, *Lamb 1343/91* (L), Mt. Ophir, *Griffith 5293* (K ), Penang, *Maingay 1667* (K), Perak, *Curtis* *3291* (K ), *O’ Brien s.n.* (BM), *Wray 464* (K ), *Scortechini 386b* (K), Sarawak, *Paul Chai S. 18464* (K), *Othman & Munting S.* *54370* (L), *Brooke 8616* (L), Selangor, *Smith BE* *103* (K ), Pahang, *Henderson 11113* (K ), *Lewis 165* (K ), *Ridley s.n.* (K ), *Wray & Robinson 5308* (BM).**Thailand:** Pattani, *Kerr 076* (K, L), Kao Luang, *Kerr 0588* (K), Doi suthep, *Hans Banziger 559* (K), Nakhon Si Thammarat, *Van Beusekom & Phengkhlai 859* (K, L). *Cult. in Hort. Bot. Bog. ex Tjianpea 135* (L), *Cult. in Hort. Bot. Bog. ex Tjianpea 351* (L). ***Tainia trinervis*** Rchb. f., **Indonesia:** Java, *Korthals s.n.* (L). **Papua New Guinea:** Koitaki Plantation, *Carr 10162* (K, BM, L), Madang, *Sohmer & Katik, LAE 75149* (K), Morobe, *Streimann* *& Kairo 45249* (K, L), *Katik* *& Croft 70751* (L), Apo *& Umbas for Dockrill 34018* (K, L). Southern Highlands, *Reeve 5393* (K), *Reeve 5570* (E, K, L), West Sepik, *Reeve 3933* (K). *Cult. in Hort. Bot. Bog. ex Tjianpea 136* (L), *Cult. in Hort. Bot. Bog. ex Tjianpea 398* (L). ***Tainia vegetissima*** Ridl., **Malaysia:** Pahang, *Ridley 16143* (K), *Wray & Robinson 5314* (K, BM), Sarawak, Mt. Dulit, *Synge S. 434* (K), Sabah, Mt. Kinabalu, *Clemens 32769* (BM). ***Tainia wrayana*** (Hook. f.) J.J. Sm., **Malaysia:** Perak, *Wray 235* (K, SING), *Wray 4059* (BM), Selongor, *Scoepadmo s.n.* (K), *Stone 5568* (K). **Indonesia:** Sumatra, *Van Steenis 9216* (L). **Thailand:** Khao Yai, *Cumberlege 1058* (K), Takaun Tai? *Kerr 0167* (K), Bought in Market Chatuchak, *Comber 1713* (K). |
