# Supplementary material for: Re-Establishment of the Genus Ania Lindl. (Orchidaceae)
Source: PLoS One. 2014 Jul 21;9(7):e103129. doi: 10.1371/journal.pone.0103129 (PMC4105443; doi:10.1371/journal.pone.0103129)
Supplement: Table S4 — Voucher information for representative species included in palynological and cytological study. The number after the second hyphen in a voucher, e.g., “−1” indicates the individual in a sampled population. (DOC) [file pone.0103129.s007.doc]

**Table S4.** **Voucher information for representative species included in palynological and cytological study. The number after the second hyphen in a voucher, e.g., “-1” indicates the individual in a sampled population.**

| ***Ania angustifolia*** Lindl., Yunnan, China, *L. Li 064* (IBSC); ***Ania hongkongensis*** (Rolfe) T. Tang & F.T.Wang, Guangdong, China, *L. Li 099* (IBSC); ***Ania penangiana*** (Hook. f.) Summerh., Hainan, China, *L. Li 113* (IBSC); ***Ania ruybarrettoi*** S.Y. Hu & Barretto, Hainan, China, *L. Li 059*(IBSC); ***Ania viridifusca*** (Hook.) Tang & F.T.Wang ex Summerh., Yunnan, China, *L. Li 069* (IBSC); ***Collabium chinense*** (Rolfe) Tang & F.T. Wang, Guangdong, China, *J.L.Zuo 21* 973(IBSC); ***Nephelaphyllum pulchrum*** Bl., Hainan, China, *L. Li 119* (IBSC); ***Tainia cordifolia*** Hook. f., Guangdong, China, *X.R.Liang 60* 691(IBSC); ***Tainia dunnii*** Rolfe -1, Guangdong, China, *L. Li 118* (IBSC); ***Tainia dunnii*** Rolfe -2, Hainan, China, *L. Li 058* (IBSC); ***Tainia latifolia*** (Lindl.) Rchb. f., Yunnan, China, *L. Li 110* (IBSC); ***Tainia*** ***macrantha*** Hook. f., Guangdong, China, *L. Li 093* (IBSC); ***Tainia minor*** Hook. f., Yunnan, China, *H. Jiang 03434* (YAF). |
| --- |
