# Supplementary material for: Re-Establishment of the Genus Ania Lindl. (Orchidaceae)
Source: PLoS One. 2014 Jul 21;9(7):e103129. doi: 10.1371/journal.pone.0103129 (PMC4105443; doi:10.1371/journal.pone.0103129)
Supplement: Table S5 — Character matrix of taxa in cladistic analysis of Tainia and its related genera. ? = missing. (DOC) [file pone.0103129.s008.doc]

**Table S5.** **Character matrix of taxa in cladistic analysis of *Tainia* and its related genera. ? = missing.**

|  | 1 2 3 4 5 |
| --- | --- |
| Taxon | 12345678901234567890123456789012345678901234567890 |
| *Ania angustifolia* | 01221002111002100211000211000110120010110000111111 |
| *Ania elmeri* | 0122100211100210021100021100011012001011000011111? |
| *Ania hongkongensis* | 01221002111002100211000211000110020010110000111111 |
| *Ania penangiana* | 01221002111002100211000211000110120010110000111111 |
| *Ania ponggolensis* | 0031010211000111111100012121101101011101000101111? |
| *Ania ruybarrettoi* | 01221002111002100211000211000110120010110000111111 |
| *Ania viridifusca* | 01221002111002100211000211000110120010110000111111 |
| *Chrysoglossum assamicum* | 0101000211000300021100011002101222211101111100000? |
| *Chrysoglossum ornatum* | 0101000211000300021100011002101222211101111100000? |
| *Collabium chinense* | 00000002100102100111100101121011110211111011000000 |
| *Collabium delavayi* | 0000000210010210011110010112101111021111101100000? |
| *Collabium formosanum* | 00000002100102100211100101121011110211111011000000 |
| *Diglyphosa latifolia* | 0101000211000300021100001102101000100110000100000? |
| *Hancockia uniflora* | 0010001000011000000100011102120012002011000001100? |
| *Nephylaphyllum tenuiflorum* | 00100010000110000110000010000120010000110000111100 |
| *Nephylaphyllum pulchrum* | 00110010000110000110000010000120010000110000111100 |
| *Tainia bicornis* | 0011000111000200021100011011101011011100000111110? |
| *Tainia cordifolia* | 00000010000100000211000120121010020110010002111100 |
| *Tainia crassa* | 0000001000010000011100012012101002011001000211110? |
| *Tainia dunnii* | 00000002110002000211000110111010110111000001111100 |
| *Tainia latifolia* | 00110001110002000211000110111010110111000001111100 |
| *Tainia laxiflora* | 0000000211010200021100011011101011011100000111110? |
| *Tainia longiscapa* | 0011101000001100011100001012101011010100000111110? |
| *Tainia macrantha* | 00000001110002000211000120121010120110010002111100 |
| *Tainia maingayi* | 0000000211000200021101021011101012011100000111111? |
| *Tainia marmorata* | 0000001000010000011100012012101002011001000211110? |
| *Tainia megalantha* | 0000001000010000011100012012101002011001000211110? |
| *Tainia minor* | 00100002110002000211000110111010110111000001111100 |
| *Tainia obpandurata* | 0000000211000200021100011011101001011100000111110? |
| *Tainia paucifolia* | 0000000211000210021100011011101011011100000111110? |
| *Tainia papuana* | 0000001000010000011100012012101002011001000211110? |
| *Tainia purpureifolia* | 0010001000011100011100001012101001010100000011110? |
| *Tainia scapigera* | 0000001000010000011100012012101002011001000211110? |
| *Tainia speciosa* | 0000000211000200021101021011101002011100000111110? |
| *Tainia trinervis* | 0000000211000200021100011011101011011100000111110? |
| *Tainia vegetissima* | 0010001000011100011100001012101001010100000011110? |
| *Tainia wrayana* | 0000001000010000011100012012101002011001000211110? |
| *Eria corneri* | 1022112011000201111100110121101011010011000101110? |
| *Eria ferruginea* | 1002012011000201111100100101101000010011000101110? |
